# Association between Opioid–Benzodiazepine Trajectories and Injurious Fall Risk among US Medicare Beneficiaries

**DOI:** 10.3390/jcm13123376

**Published:** 2024-06-07

**Authors:** Grace Hsin-Min Wang, Juan M. Hincapie-Castillo, Walid F. Gellad, Bobby L. Jones, Ronald I. Shorr, Seonkyeong Yang, Debbie L. Wilson, Jeannie K. Lee, Gary M. Reisfield, Chian K. Kwoh, Chris Delcher, Khoa A. Nguyen, Christopher A. Harle, Zachary A. Marcum, Wei-Hsuan Lo-Ciganic

**Affiliations:** 1 Department of Pharmaceutical Outcomes and Policy, College of Pharmacy, University of Florida, Gainesville, FL 32610, USA; hsinminwang@ufl.edu (G.H.-M.W.); bobby.jones@ufl.edu (B.L.J.); yang.se@ufl.edu (S.Y.); debbie.wilson@ufl.edu (D.L.W.); 2 Department of Epidemiology, Gillings School of Global Public Health, University of North Carolina at Chapel Hill, Chapel Hill, NC 27599, USA; jhincapie-castillo@unc.edu; 3 Injury Prevention Research Center, University of North Carolina at Chapel Hill, Chapel Hill, NC 27599, USA; 4 Center for Pharmaceutical Policy and Prescribing, Health Policy Institute, University of Pittsburgh, Pittsburgh, PA 15261, USA; walid.gellad@pitt.edu; 5 Department of Medicine, School of Medicine, University of Pittsburgh, Pittsburgh, PA 15213, USA; 6 Center for Health Equity Research Promotion, Veterans Affairs Pittsburgh Healthcare System, Pittsburgh, PA 15240, USA; 7 North Florida/South Georgia Veterans Health System Geriatric Research Education and Clinical Center, Gainesville, FL 32608, USA; rshorr@ufl.edu; 8 Department of Pharmacy Practice and Science, College of Pharmacy, University of Arizona, Tucson, AZ 85724, USA; jklee@arizona.edu; 9 Divisions of Addiction Medicine & Forensic Psychiatry, Departments of Psychiatry & Anesthesiology, College of Medicine, University of Florida, Gainesville, FL 32611, USA; garyr@ufl.edu; 10 University of Arizona Arthritis Center, College of Medicine, University of Arizona, Tucson, AZ 85721, USA; kwoh@arizona.edu; 11 Division of Rheumatology, College of Medicine, University of Arizona, Tucson, AZ 85724, USA; 12 Pharmacy Practice & Science, Institute for Pharmaceutical Outcomes & Policy, College of Pharmacy, University of Kentucky, Lexington, KY 40506, USA; chris.delcher@uky.edu; 13 Department of Pharmacotherapy & Translational Research, College of Pharmacy, University of Florida, Gainesville, FL 32610, USA; nguyen.khoa@ufl.edu; 14 Department of Health Policy and Management, School of Public Health, Indiana University, Indianapolis, IN 47405, USA; charle@iu.edu; 15 School of Pharmacy, University of Washington, Seattle, WA 98195, USA; zach.marcum@gmail.com

**Keywords:** trajectory, opioid, benzodiazepine, falls, fractures

## Abstract

**Background/Objectives:** Concurrent opioid (OPI) and benzodiazepine (BZD) use may exacerbate injurious fall risk (e.g., falls and fractures) compared to no use or use alone. Yet, patients may need concurrent OPI-BZD use for co-occurring conditions (e.g., pain and anxiety). Therefore, we examined the association between longitudinal OPI-BZD dosing patterns and subsequent injurious fall risk. **Methods:** We conducted a retrospective cohort study including non-cancer fee-for-service Medicare beneficiaries initiating OPI and/or BZD in 2016–2018. We identified OPI-BZD use patterns during the 3 months following OPI and/or BZD initiation (i.e., trajectory period) using group-based multi-trajectory models. We estimated the time to first injurious falls within the 3-month post-trajectory period using inverse-probability-of-treatment-weighted Cox proportional hazards models. **Results:** Among 622,588 beneficiaries (age ≥ 65 = 84.6%, female = 58.1%, White = 82.7%; having injurious falls = 0.45%), we identified 13 distinct OPI-BZD trajectories: Group (A): Very-low OPI-only (early discontinuation) (44.9% of the cohort); (B): Low OPI-only (rapid decline) (15.1%); (C): Very-low OPI-only (late discontinuation) (7.7%); (D): Low OPI-only (gradual decline) (4.0%); (E): Moderate OPI-only (rapid decline) (2.3%); (F): Very-low BZD-only (late discontinuation) (11.5%); (G): Low BZD-only (rapid decline) (4.5%); (H): Low BZD-only (stable) (3.1%); (I): Moderate BZD-only (gradual decline) (2.1%); (J): Very-low OPI (rapid decline)/Very-low BZD (late discontinuation) (2.9%); (K): Very-low OPI (rapid decline)/Very-low BZD (increasing) (0.9%); (L): Very-low OPI (stable)/Low BZD (stable) (0.6%); and (M): Low OPI (gradual decline)/Low BZD (gradual decline) (0.6%). Compared with Group (A), six trajectories had an increased 3-month injurious falls risk: (C): HR = 1.78, 95% CI = 1.58–2.01; (D): HR = 2.24, 95% CI = 1.93–2.59; (E): HR = 2.60, 95% CI = 2.18–3.09; (H): HR = 2.02, 95% CI = 1.70–2.40; (L): HR = 2.73, 95% CI = 1.98–3.76; and (M): HR = 1.96, 95% CI = 1.32–2.91. **Conclusions:** Our findings suggest that 3-month injurious fall risk varied across OPI-BZD trajectories, highlighting the importance of considering both dose and duration when assessing injurious fall risk of OPI-BZD use among older adults.

## 1. Introduction

With the global population aging, injurious falls (e.g., falls and fractures) have become a significant public health issue [1]. Each year, 25% of older people in the United States (US) report ≥1 fall of which 37% cause serious injuries that require medical treatment or restrict patients’ activity for >1 day [2,3]. Injurious falls increase the risks of emergency department (ED) visits, hospitalizations, and death, which can lead to high medical costs [1]. As of May 2024, the Centers for Disease Control and Prevention estimated that 36 million older adults fall each year, resulting in 32,000 deaths, 3 million ED visits, 800,000 hospitalizations, and USD 50 billion in medical costs using the latest data from 2020 to 2021 [2,3].

According to a systemic review and meta-analysis, opioid (OPI) use and benzodiazepine (BZD) use were both associated with an increased injurious fall risk compared to no use, with an odds ratio (OR) of 1.60 (95% confidence interval [95% CI] = 1.35–1.91) and 1.42 (95% CI = 1.22–1.65), respectively [4,5]. Concurrent use of OPIs and BZDs (OPI-BZD) may further exacerbate the risk of injurious falls. For example, a retrospective cohort study found that for older adults receiving BZD monotherapy, having OPI use within 180 days before BZD initiation was associated with an increased injurious fall risk (hazard ratio [HR] = 1.22, 95% CI = 1.07–1.40) [6]. Therefore, the Beers criteria recommended against the concurrent use of OPI and BZD [7]. Despite these warnings, OPI is sometimes co-prescribed with BZD [8]. In 2015, the co-prescribing rate of OPI and BZD was roughly 2.7% [8].

Existing evidence assessing OPI-BZD’s associated risk of injurious falls is limited by (1) reporting the cross-sectional co-prescription rate of OPI and BZD, (2) using an intention-to-treat design (e.g., one is considered an OPI user if prescribed with an OPI at the beginning of the study), and (3) relying on a simplistic definition of concurrent use (e.g., >30 overlapping days of supply) [9]. However, OPI and BZD may be used intermittently, and doses may change over time [10,11]. Thus, a better understanding of the differential risk of injurious falls among distinct longitudinal dose/duration patterns of OPI-BZD use (i.e., trajectories) is needed. In this study, we aimed to investigate the OPI-BZD trajectories most associated with injurious falls.

## 2. Materials and Methods

### 2.1. Data Sources and Study Design

This retrospective cohort study used a 15% nationally representative sample of Medicare beneficiaries plus all beneficiaries in Florida from 2016 to 2018. Medicare is a national health insurance program available to US populations aged 65+ and those with end-stage renal disease or disability. We restricted our analysis to beneficiaries enrolled in fee-for-service plans including Part A (hospital), Part B (medical), and Part D (prescription drug) as previous reports suggested there is incomplete data capture for those enrolled in Medicare Part C plans (Medicare Advantage) [12].

Throughout the study period, we excluded beneficiaries who (1) were non-US residents, (2) had malignant cancer diagnoses, (3) had claims for hospice services, and (4) did not have any eligible OPI or BZD prescriptions. Eligible OPI was defined as non-injectable, non-buprenorphine (for opioid use disorder) OPI. Then, among beneficiaries who received ≥1 eligible OPI or BZD from 2016 to 2018, we further excluded those who (1) did not have continuous fee-for-service enrollment 6 months prior to and 3 months after the index date (i.e., first OPI or BZD dispensing date); (2) had their first OPI or BZD prescription dispensed before 1 July 2016, or after 1 October 2018; (3) had any fall-related diagnosis or procedure codes within 6 months before the index date; (4) had their first OPI or BZD prescription dispensed on the same date of injurious falls claims; (5) had claims for accidental injury within 6 months before the index date as they were at a higher risk of having injurious falls; or (6) had claims for orthopedic visits with any related image diagnosis (+/−3 days) within 1 month before the index date to avoid misclassification of an exposure-outcome temporal relationship (Supplementary Figure S1).

This study followed the Strengthening the Reporting of Observational Studies in Epidemiology (STROBE) guideline and was approved by the University of Florida Institutional Review Board [13].

### 2.2. Exposure Ascertainment

The exposure of interest was the patient’s membership in a distinct trajectory of OPI-BZD use. We used the data-driven group-based multi-trajectory modeling (GBMTM) approach to identify subgroups following similar medication use patterns over time (i.e., trajectories) [14,15,16,17]. GBMTM estimates the maximum likelihood of each patient’s membership in different trajectory groups [16]. In GBMTM, the dependent variable was the daily measure of average standardized daily dose (SDD) for OPIs and BZDs; the independent variable was the 3-month trajectory measurement period after the index date (Supplementary Figure S2). The SDD for OPIs was converted into the average daily morphine milligram equivalents (MME) using the dispensing information (i.e., fill date, dose, and days of supply) and the conversion factors provided by the CDC [18,19]. The SDD for BZDs was the daily diazepam milligram equivalents (DME) calculated based on published equivalent dosing conversions (Table S1). Analytical details regarding the identification of distinct OPI-BZD trajectories were described in our previous work.

We allowed for the most flexible functional form of time (up to the fifth-order polynomial function). The final number of trajectories was selected using a combination of criteria, including (1) larger Bayesian Information Criterion [20]; (2) Nagin’s criteria [21]; (3) requirement of each trajectory group to have at least 2 injurious falls (to obtain valid risk estimates in Cox models) [22]; and (4) requirement of each trajectory to have sufficient number of beneficiaries to support clinical relevance of identified patterns, with a preference for fewer trajectories to minimize complexity and maintain clinical interpretability. The study investigators qualitatively labeled individual trajectories based on the observed dose/duration patterns for OPI and BZD use over the trajectory measurement period. In general, we defined OPI dose level as very-low- (SDD < 25 MME), low- (25–50 MME), moderate- (51–90 MME), high- (91–150 MME), and very-high-dose (>150 MME) [23]. Similarly, we defined BZD dose level as very-low- (<10 DME), low- (10–20 DME), moderate- (21–40 DME), high- (41–60 DME), and very-high-dose (>60 DME). If discontinuation of OPIs or BZDs occurred within 30 days after initiation, we defined it as early discontinuation; otherwise, it was defined as late discontinuation. If drug dose was reduced by >10 MME or >10 DME within 30 days, then the reduction was defined as rapid decline; otherwise, we defined it as gradual decline.

### 2.3. Outcome Ascertainment

The primary outcome was time to a patient’s first injurious falls recorded in the 3 months following the index date. Patients were censored at the earliest outcome occurrence, if they switched to a Medicare Advantage plan or died, or at the end of the study period. We used International Classification of Diseases codes (ICD) to identify injurious falls based on validated algorithms (Table S2) [24,25,26]. We chose 3 months as trajectory and outcome measurement windows because injurious falls are more likely to occur due to changes in drug use (e.g., new addition, dose change). We also examined the 6-month risk of injurious falls after the index date in a sensitivity analysis to test the robustness of the results.

### 2.4. Covariate Ascertainment

We measured relevant covariates during the 6 months prior to the index date. Demographic covariates included age, sex, race/ethnicity, disability status, receipt of low-income subsidy (LIS), dual Medicaid eligibility, and metropolitan residence. Based on the literature and clinician inputs [27,28,29], covariates related to health status included Elixhauser comorbidity index, substance use disorders (SUD), anxiety, mood, sleep, musculoskeletal disorders, and individual pain conditions (Table S2). Health services use factors included any hospitalization, number of ED visits, and number of outpatient visits. We also measured a series of medication-use-related variables including number of antidepressants, antipsychotics, gabapentinoids, muscle relaxants, naltrexone, and polypharmacy (i.e., ≥5 medications in total). Finally, we described several OPI/BZD-related characteristics including type of medications (e.g., short-acting, long-acting), unique medication ingredients, average days of supply and average number of fills for OPI and BZD prescriptions in the 3-month trajectory measurement period, respectively.

### 2.5. Statistical Analysis

We used the inverse probability of treatment weighting (IPTW) approach to minimize the confounding by different patient characteristics and disease complexity across trajectory groups. First, we included baseline covariates measured in the 6 months before the index date in the gradient boosting machine to estimate the probability of an individual’s likelihood of being grouped in a specific trajectory (i.e., propensity score [PS]). IPTW was calculated as the 1/PS. We compared patient characteristics across trajectory groups before and after IPTW using the absolute standardized mean differences (ASMD) [30]. Then, we applied the IPTW in the weight function of the Cox proportional hazards model to compare the time-to-event within 3 months after the index date across different trajectories. Covariates with non-negligible differences (mean ASMD > 0.1) after IPTW were also adjusted in the model (i.e., doubly robust method) [31]. We examined the proportional hazards assumption using Schoenfeld residuals.

We reported patient characteristics using numbers and proportions for categorical variables and mean and standard deviation (SD) for continuous variables. To assess the risk of injurious falls, we reported number of injurious falls, follow-up duration, and crude/adjusted HR with 95% CI. Finally, to estimate the potential effects of unmeasured confounder on our findings, we calculated E-values to assess the minimum strength of association a confounder would need to have with the exposure (i.e., trajectory membership) and outcome (i.e., injurious falls) to bias away the observed association, conditional on measured covariates. A larger E-value indicated that the association between the exposure and outcome was more robust than the unmeasured confounders [32].

We used STATA 16.0 (Stata-Corp LP, College Station, TX, USA) and the TRAJ macro (http://www.andrew.cmu.edu/user/bjones; accessed on 1 May 2024) for GBMTM; R packages, tableone and survey for ASMD; and SAS version 9.4 (SAS Inc., Cary, NC, USA) for other analyses.

## 3. Results

**Figure 1:**
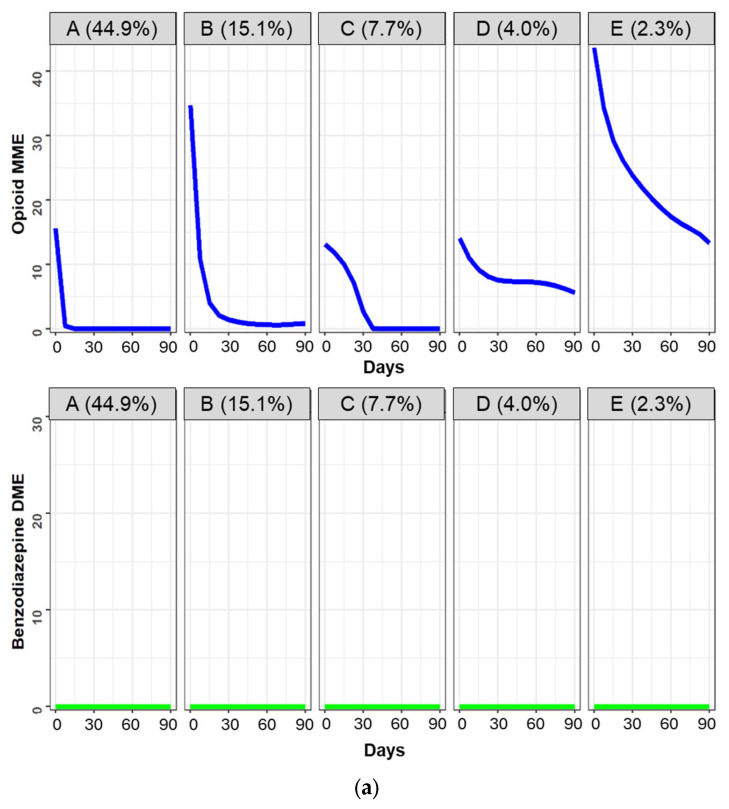

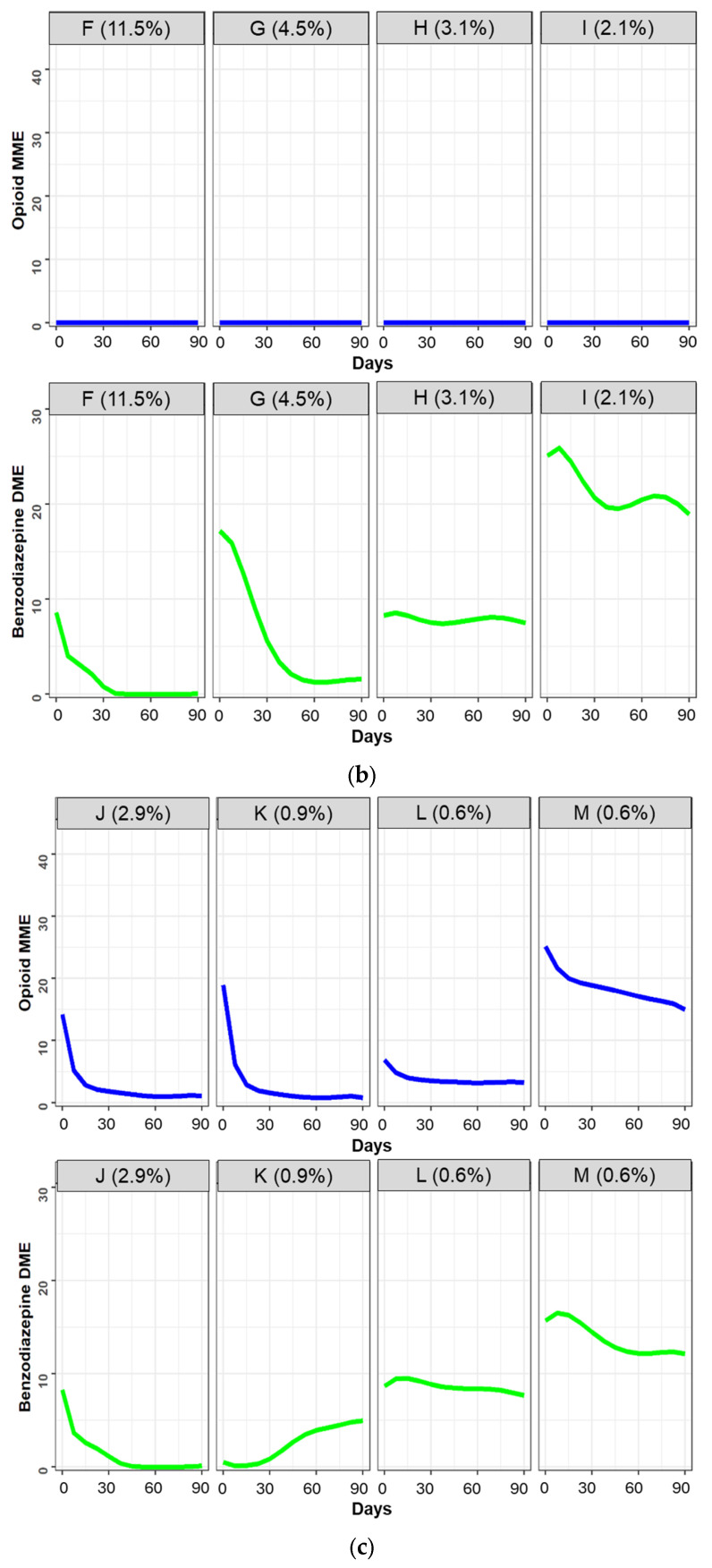
Trajectories of opioid and benzodiazepine utilization patterns among 622,588 Medicare beneficiaries. Trajectory groups can be divided into OPI use only (**a**), BZD use only (**b**), and OPI and BZD use (**c**). (**a**) shows A: Very-low OPI-only (early discontinuation) (*n* = 279,263; 44.9%); B: Low OPI-only (rapid decline) (*n* = 93,703; 15.1%); C: Very-low OPI-only (late discontinuation) (*n* = 47,851; 7.7%); D: Low OPI-only (gradual decline) (*n* = 24,952; 4.0%); E: Moderate OPI-only (rapid decline) (*n* = 14,225; 2.3%). (**b**) shows F: Very-low BZD-only (late discontinuation) (*n* = 71,715; 11.5%); G: Low BZD-only (rapid decline) (*n* = 28,109; 4.5%); H: Low BZD-only (stable) (*n* = 19,230; 3.1%); I: Moderate BZD-only (gradual decline) (*n* = 13,013; 2.1%). (**c**) shows J: Very-low OPI (rapid decline)/Very-low BZD (late discontinuation) (*n* = 17,750; 2.9%); K: Very-low OPI (rapid decline)/Very-low BZD (increasing) (*n* = 5601; 0.9%); L: Very-low OPI (stable)/Low BZD (stable) (*n* = 3729; 0.6%); M: Low OPI (gradual decline)/Low BZD (gradual decline) (*n* = 3447; 0.6%). We calculated SDDs for OPIs using MME and for BZDs using DME. To facilitate the labeling of opioid and benzodiazepine dose levels for each trajectory, we defined opioid dosage use as very-low- (SDD < 25 MME), low- (25–50 MME), moderate- (51–90 MME), high- (91–150 MME), and very-high-dose (>150 MME). Similarly, we defined BZD dosage use as very-low- (<10 DME), low- (10–20 DME), moderate- (21–40 DME), high- (41–60 DME), and very-high-dose (>60 DME). Abbreviations: BZD, benzodiazepine; DME, diazepam milligram equivalent; MME, morphine milligram equivalent; OPI, opioid; SDD, standardized daily dose.

Figure 1 illustrates the daily dose utilization patterns for OPI and BZD use in the 3-month period following OPI or BZD initiation. Based on the labeling rules described in the Methods, we identified 13 distinct OPI-BZD trajectories. Figure 1a shows five trajectory groups with OPI use only (*n* = 459,994; 73.9% of the cohort) including Group (A): Very-low OPI-only (early discontinuation) (*n* = 279,263; 44.9%); (B): Low OPI-only (rapid decline) (*n* = 93,703; 15.1%); (C): Very-low OPI-only (late discontinuation) (*n* = 47,851; 7.7%); (D): Low OPI-only (gradual decline) (*n* = 24,952; 4.0%); (E): Moderate OPI-only (rapid decline) (*n* = 14,225; 2.3%). Figure 1b shows 4 trajectory groups using BZD only (*n* = 132,067; 21.2%) including (F): Very-low BZD-only (late discontinuation) (*n* = 71,715; 11.5%); (G): Low BZD-only (rapid decline) (*n* = 28,109; 4.5%); (H): Low BZD-only (stable) (*n* = 19,230; 3.1%); (I): Moderate BZD-only (gradual decline) (*n* = 13,013; 2.1%). Figure 1c shows 4 trajectory groups with OPI-BZD use (*n* = 30,527; 4.9%) including (J): Very-low OPI (rapid decline)/Very-low BZD (late discontinuation) (*n* = 17,750; 2.9%); (K): Very-low OPI (rapid decline)/Very-low BZD (increasing) (*n* = 5601; 0.9%); (L): Very-low OPI (stable)/Low BZD (stable) (*n* = 3729; 0.6%); and (M): Low OPI (gradual decline)/Low BZD (gradual decline) (*n* = 3447; 0.6%).

Before IPTW, there were multiple non-negligible differences in patient characteristics across trajectory groups. After IPTW, all characteristics had a mean ASMD of <0.1, indicating a balance across trajectories (Table 1). Table S3 includes detailed patient characteristics in each trajectory. Table S4 presents the minimum and maximum ASMDs across the 78 group comparisons ($C_{2}^{13}$).

As shown in Table S5, the most common OPI was short-acting hydrocodone (45.8%), which was similar across trajectory groups. During the 3-month trajectory measurement period, the mean days of supply was 8.4 (SD = 9.4), the mean SDD was 2.7 (SD = 5.1), and the mean number of OPI fills was 1.4 (SD = 0.9). Table S6 suggested that the most widely used BZD was alprazolam (34.6%). The mean days of supply was 24.2 (SD = 22.5), the mean SDD was 1.3 (SD = 4.2), and the mean number of fills was 1.5 (SD = 1.0). Group F and Group J had lower mean days of supply (F: 14.6 [SD = 11.7], J: 9.8 [SD = 10.6]) and mean number of fills (F: 1.1 [SD = 0.3], J: 1.1 [SD = 0.3]) compared to the overall cohort.

**Figure 2:**
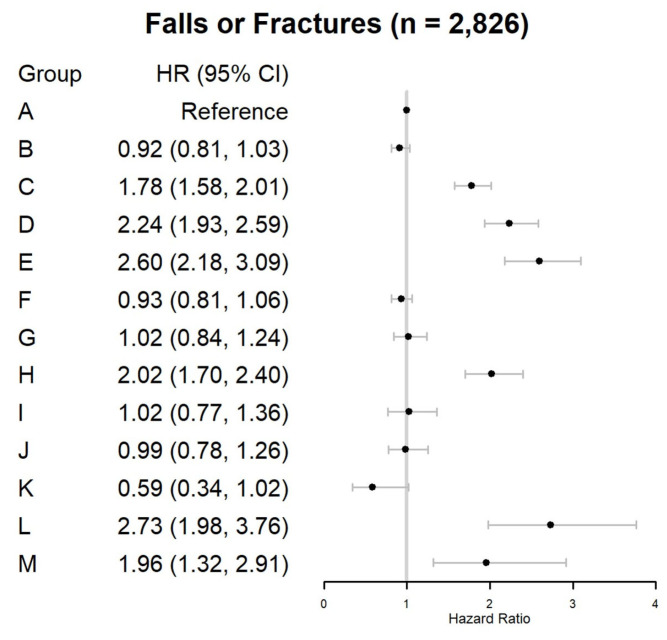
Trajectories of opioid and benzodiazepine utilization patterns and risk of injurious falls during the 3-month trajectory period. Trajectory groups include A: Very-low OPI-only (early discontinuation) (*n* = 279,263; 44.9%); B: Low OPI-only (rapid decline) (*n* = 93,703; 15.1%); C: Very-low OPI-only (late discontinuation) (*n* = 47,851; 7.7%); D: Low OPI-only (gradual decline) (*n* = 24,952; 4.0%); E: Moderate OPI-only (rapid decline) (*n* = 14,225; 2.3%); F: Very-low BZD-only (late discontinuation) (*n* = 71,715; 11.5%); G: Low BZD-only (rapid decline) (*n* = 28,109; 4.5%); H: Low BZD-only (stable) (*n* = 19,230; 3.1%); I: Moderate BZD-only (gradual decline) (*n* = 13,013; 2.1%); J: Very-low OPI (rapid decline)/Very-low BZD (late discontinuation) (*n* = 17,750; 2.9%); K: Very-low OPI (rapid decline)/Very-low BZD (increasing) (*n* = 5601; 0.9%); L: Very-low OPI (stable)/Low BZD (stable) (*n* = 3729; 0.6%); M: Low OPI (gradual decline)/Low BZD (gradual decline) (*n* = 3447; 0.6%). We calculated SDDs for OPIs using MME and for BZDs using DME. To facilitate the labeling of opioid and benzodiazepine dose levels for each trajectory, we defined opioid dosage use as very-low- (SDD < 25 MME), low- (25–50 MME), moderate- (51–90 MME), high- (91–150 MME), and very-high-dose (>150 MME). Similarly, we defined BZD dosage use as very-low- (<10 DME), low- (10–20 DME), moderate- (21–40 DME), high- (41–60 DME), and very-high-dose (>60 DME). Abbreviations: BZD, benzodiazepine; DME, diazepam milligram equivalent; MME, morphine milligram equivalent; OPI, opioid; SDD, standardized daily dose.

In the 3 months after OPI or BZD initiation, 2826 (0.45%) beneficiaries experienced injurious falls (Table 2 and Figure 2). Compared with Group (A) (crude rate: 1037 per 10,000 person-months), the 3-month injurious falls risk significantly increased among individuals in the six trajectories (18.2% of the cohort): (C) HR = 1.78, 95% CI = 1.58–2.01, (D) HR = 2.24, 95% CI = 1.93–2.59, (E) HR = 2.60, 95% CI = 2.18–3.09, (H) HR = 2.02, 95% CI = 1.70–2.40, (L) HR = 2.73, 95% CI = 1.98–3.76, and (M) HR = 1.96, 95% CI = 1.32–2.91.

The results were generally robust in the sensitivity analysis where the risk of injurious falls within 6 months after the first prescription of OPI or BZD was evaluated (Table S7 and Figure S3). In addition to Groups (C) HR = 1.54, 95% CI = 1.40–1.69, (D) HR = 1.93, 95% CI = 1.72–2.16, (E) HR = 2.22, 95% CI = 1.93–2.55, (H) HR = 1.82, 95% CI = 1.59–2.07, (L) HR = 2.64, 95% CI = 2.08–3.36, (M) HR = 1.72, 95% CI = 1.26–2.35, Group (I) HR = 1.31, 95% CI = 1.09–1.58 also had an increased risk compared to Group (A).

Table S8 shows our findings’ robustness to potential influences of unmeasured confounders. The large E–values for the HRs of trajectory groups C, D, E, H, L, and M ranged from 3.48 to 6.42, indicating that estimated HRs were robust to unmeasured confounders.

## 4. Discussion

To our knowledge, this study is the first to identify longitudinal trajectories of concurrent OPI and BZD use at a high risk of injurious falls. Our findings aligned with previous studies showing that the effect of OPI and BZD on injurious fall risk may be dose-dependent. For OPI, Miller et al., divided all OPI prescriptions into low- (1–75 mg), med- (76–225 mg), and high-dose (>225 mg) by the equivalents of codeine per day, and they found a dose–response relationship between OPI use and risk of fractures (low-dose HR = 2.2, 95% CI = 0.9–5.2; medium-dose HR = 4.6, 95% CI = 3.2–6.6; high-dose HR = 5.1, 95% CI = 3.7–7.2) [33]. Saunders et al., divided OPI use into low- (1–19 mg), med- (20–50 mg) and high-dose (>50 mg) by its daily MME dose, and they found a dose-response relationship between OPI use and fracture risk (low-dose HR = 1.20, 95% CI = 0.92–1.56; med-dose HR = 1.34, 95% CI = 0.89–2.01; high-dose HR = 2.00, 95% CI = 1.24–3.24) [34]. For BZD, Ray et al., found that the rate ratio of injurious fall risk increased from 1.30 (95% CI = 1.12–1.52) for a DME of ≤2 mg, to 2.21 (95% CI = 1.89–2.60) for a DME of >8 mg compared to no use [35]. Long-term drug use may also be associated with an increase in the risk of injurious falls. For example, a nationwide population-based case-crossover study suggested that the longer the prescription period of BZDs, the higher the risk of injurious falls (day 7 OR = 1.48, 95% CI = 1.14–1.93, day 49 OR = 2.02, 95% CI = 1.64–2.49, day 120 OR = 3.22, 95% CI = 2.58–4.02) [36]. However, the dose and duration of OPI-BZD use are likely to change over time and vary by patient characteristics, affecting injurious fall risk. Unlike prior studies using simple concurrent use measures (e.g., any overlapping use) and intention-to-treat exposure assignment, our study sheds additional insights into the longitudinal use patterns of OPI-BZD that are most associated with injurious fall risk.

This study has several limitations when interpreting the study findings. First, we used claims data, which provided limited information regarding disease severity (e.g., chronic pain, anxiety), actual drug-taking behavior, and some self-paid medications (e.g., over-the-counter analgesics). These unmeasured confounders may result in residual confounding, yet our E-value proved that the risk estimates were robust. We also adjusted for several clinical diagnoses and medication use as a proxy of these unmeasured confounders. Second, a follow-up period of 3 months after the first prescription of OPI and/or BZD may be too short to identify all injurious falls. However, the high-risk groups remained similar in the sensitivity analysis lengthening the follow-up period to 6 months. Third, we presented the average OPI-BZD dosing patterns in each trajectory, which may vary from individuals among each trajectory. However, GBMTM allows us to assign patients with the most similar dosing patterns into a group to provide insights on medication use patterns in real-world clinical settings. Fourth, our findings may not be generalized to other populations (e.g., Medicaid).

In conclusion, among the 13 OPI–BZD trajectories during the 3 months following OPI or BZD initiation, trajectories with concurrent use of OPI and BZD were not necessarily associated with a higher risk of injurious falls. Instead, those with a higher dose of OPI (≥25 MME) and/or BZD (≥10 DME) or with a longer duration of use were associated with a 1.8 to 2.7 times increased injurious fall risk compared to the lowest dose (<25 MME), early discontinuing, OPI-only trajectory. Therefore, both dose and duration rather than simple concurrent use of OPI and BZD impact the risk of injurious falls. Our findings have significant clinical implications. For example, though the current CDC guidelines recommend against concurrent OPI–BZD use [37], it may not be clinically actionable to completely avoid co-prescribing OPI and BZD for patients in need (e.g., those suffering from co-occurring severe anxiety and chronic pain), since the benefits may outweigh the risk of injurious falls. Identifying trajectories associated with a higher risk of injurious falls provides additional information for clinicians when prescribing OPI and/or BZD for patients. Additionally, deprescribing (e.g., dose reduction and discontinuation) of OPI and BZD is common and recommended if pain and related anxiety or sleep disorders are relieved [38]. The rate of deprescribing may affect the risk of injurious falls, yet this was not reported in the previous literature. We found that a dose reduction of >10 MME/DME within 30 days may still be safe regarding the injurious fall risk although abrupt tapering or discontinuation is not recommended due to potential withdrawal syndromes and unintended consequences [38]. Therefore, our study identified dosing patterns of OPI-BZD at a high risk of injurious falls among older adults, providing clinically valuable information for clinicians when prescribing the drugs to older adults.

## 5. Conclusions

Our findings suggest that 3-month injurious fall risk varied across OPI-BZD trajectories, highlighting the importance of considering both dose and duration when assessing fall risks of OPI-BZD use among older adults.

## Figures and Tables

**Table 1:** Characteristics of Medicare beneficiaries.

Trajectory Groups	Overall: *n* = 622,588	ASMD ^‡^	
		Before IPTW	After IPTW
Age ≥65 years, %	84.6	0.18	0.01
Female, %	58.1	0.14	0.02
Race/ethnicity group, %			
White	82.7	0.09	0.02
Black	9.0	0.10	0.03
Others	8.3	0.06	0.01
Disability status, %	21.6	0.18	0.01
LIS/Dual eligibility, %			
No LIS/dual eligibility	72.9	0.18	0.02
LIS or dual eligibility	5.1	0.09	0.01
LIS and dual eligibility	22.0	0.16	0.02
Metropolitan residence	81.9	0.07	0.02
Elixhauser Comorbidity Index, mean (SD)	3.3 (2.7)	0.14	0.01
Opioid use disorder, %	0.4	0.07	0.01
Alcohol use disorders, %	1.2	0.06	0.02
Other SUD, %	0.8	0.07	0.01
Anxiety disorders, %	11.1	0.27	0.02
Mood disorders, %	12.3	0.17	0.02
Sleep disorders, %	15.1	0.10	0.01
Musculoskeletal conditions, %	47.4	0.27	0.03
Pain conditions, %			
Osteoarthritis	36.8	0.20	0.02
Low back pain	21.2	0.23	0.02
Neck pain	8.0	0.11	0.01
Chest pain	12.4	0.06	0.01
Abdominal pain	17.5	0.11	0.01
Rheumatoid arthritis	2.6	0.07	0.01
Pelvic pain	3.1	0.05	0.01
Headache/migraine	5.2	0.06	0.01
TMJ	0.2	0.02	0.02
Others	21.5	0.11	0.01
Any hospitalization, %	13.8	0.21	0.04
ED visits, %			
0	87.9	0.09	0.01
1	10.5	0.08	0.01
≥2	1.7	0.05	0.01
Outpatient visits, %			
0	38.0	0.12	0.02
1	23.4	0.01	0.01
2–5	33.3	0.10	0.02
>5	5.3	0.08	0.01
No. antidepressants	0.8 (2.1)	0.14	0.01
No. antipsychotics	0.3 (1.7)	0.14	0.02
No. gabapentinoids	0.2 (1.0)	0.11	0.01
No. muscle relaxants	0.1 (0.6)	0.07	0.01
No. naltrexone	0.0 (0.1)	0.02	0.01
Polypharmacy, %	87.6	0.11	0.01

Abbreviations: ED, emergency department; LIS, low-income subsidy; No., number of; SD, standard deviation; SUD: substance use disorder; TMJ: temporomandibular disorder pain. ^‡^ Mean ASMD of 78 ASMDs from group comparisons (the number of 2 combinations from 13 trajectories). Table S4 includes the maximum and minimum ASMDs.

**Table 2:** Trajectories of opioid and benzodiazepine utilization patterns and 3-month risk of injurious falls among Medicare beneficiaries (*n* = 622,588).

Trajectory Groups ^§^	Injurious Falls (*n* = 2826)			
	N (Crude Rate *)	Days of Follow-Up, Median (IQR)	HR (95% CI)	
			Unadjusted	Adjusted ^†^
OPI use only				
A: Very-low OPI-only (early discontinuation)	1037 (12.4)	44 (41.0)	Reference	Reference
B: Low OPI-only (rapid decline)	323 (11.5)	51 (45.0)	0.93 (0.82, 1.05)	0.92 (0.81, 1.03)
C: Very-low OPI-only (late discontinuation)	360 (25.1)	37 (46.5)	2.03 (1.80, 2.29)	1.78 (1.58, 2.01)
D: Low OPI-only (gradual decline)	219 (29.3)	48 (37.0)	2.37 (2.05, 2.74)	2.24 (1.93, 2.59)
E: Moderate OPI-only (rapid decline)	122 (28.6)	40 (41.0)	2.32 (1.92, 2.79)	2.60 (2.18, 3.09)
BZD use only				
F: Very-low BZD-only (late discontinuation)	276 (12.8)	51 (39.5)	1.04 (0.91, 1.18)	0.93 (0.81, 1.07)
G: Low BZD-only (rapid decline)	122 (14.5)	47 (46.0)	1.17 (0.97, 1.41)	1.02 (0.84, 1.24)
H: Low BZD-only (stable)	147 (25.5)	34 (38.0)	2.06 (1.74, 2.45)	2.02 (1.70, 2.40)
I: Moderate BZD-only (gradual decline)	58 (14.9)	51 (45.0)	1.20 (0.92, 1.56)	1.03 (0.77, 1.36)
OPI and BZD use				
J: Very-low OPI (rapid decline)/ Very-low BZD (late discontinuation)	73 (13.7)	57 (36.0)	1.11 (0.87, 1.40)	0.99 (0.78, 1.26)
K: Very-low OPI (rapid decline)/ Very-low BZD (increasing)	14 (8.3)	67 (29.0)	0.67 (0.40, 1.14)	0.59 (0.34, 1.02)
L: Very-low OPI (stable)/ Low BZD (stable)	48 (42.9)	45 (51.5)	3.48 (2.61, 4.65)	2.73 (1.98, 3.76)
M: Low OPI (gradual decline)/ Low BZD (gradual decline)	27 (26.1)	41 (39.0)	2.11 (1.44, 3.10)	1.96 (1.32, 2.91)

Abbreviations: CI, confidence interval; BZD, benzodiazepines; HR, hazard ratio; OPI, opioid; OPI-BZD, concurrent opioid and benzodiazepine use. ^§^ To facilitate the labeling of opioid and benzodiazepine dose levels for each trajectory, we defined opioid dosage use as very-low- (SDD < 25 MME), low- (25–50 MME), moderate- (51–90 MME), high- (91–150 MME), and very-high-dose (>150 MME). Similarly, we defined BZD dosage use as very-low- (<10 DME), low- (10–20 DME), moderate- (21–40 DME), high- (41–60 DME), and very-high-dose (>60 DME). We considered early discontinuation of OPIs or BZDs when discontinuation occurred within 30 days after initiation; otherwise, it was defined as late discontinuation. If drug dose was reduced by >10 MME or >10 DME within 30 days, then the reduction was defined as rapid decline; otherwise, we defined it as gradual decline. * The unit for crude rates is per 10,000 person-months. ^†^ For primary analysis, we excluded 8 beneficiaries with extreme IPTWs (>10) using trimming methods to increase validity of treatment effect estimates.

## Data Availability

The datasets generated or analyzed in this study are not publicly accessible per Center’s for Medicare & Medicaid Services (CMS) regulation. Researchers wishing to analyze these datasets must submit a formal application to ResDAC. For more information, please visit their website at https://resdac.org/cms-research-identifiable-request-process-timeline (accessed on 1 March 2024).

## Supplementary Materials

The following supporting information can be downloaded at: https://www.mdpi.com/article/10.3390/jcm13123376/s1, Table S1. Equivalency Conversion Table for Benzodiazepines, Table S2. ICD-9-CM and ICD-10-CM Codes of Diseases and Conditions Used in the Study, Table S3. Characteristics of Medicare Beneficiaries by Opioid and Benzodiazepine Trajectory Group, Table S4. Minimum and Maximum Standardized Mean Differences across Trajectory Group Comparisons, Table S5. Patterns of Opioid Use During 3-month Trajectory Measurement Period by Trajectory Group, Table S6. Patterns of Benzodiazepine Use During 3-month Trajectory Measurement Period by Trajectory Group, Table S7. Trajectories of Opioid and Benzodiazepine Use and Risk of Subsequent Injurious Falls among Medicare Beneficiaries, Table S8. E-values of Hazard Ratio Estimates for Injurious Falls among Medicare Beneficiaries, Figure S1. Sample Size Flowchart, Figure S2. Study Design Diagram, Figure S3. Trajectories of Opioid and Benzodiazepine Utilization Patterns and Risk of Injurious Falls: Sensitivity Analyses including Beneficiaries with Injurious Falls During the 6-month Trajectory Period (*n* = 622,588).
