# Supplementary material for: Association between Opioid–Benzodiazepine Trajectories and Injurious Fall Risk among US Medicare Beneficiaries
Source: J Clin Med. 2024 Jun 7;13(12):3376. doi: 10.3390/jcm13123376 (PMC11204130; doi:10.3390/jcm13123376)
Supplement: Supplementary file 1 [file jcm-13-03376-s001.zip › jcm-2985037-supplementary.pdf]

Supplement

Table S1. Equivalency Conversion Table for Benzodiazepines
Table S2. ICD-9-CM and ICD-10-CM Codes of Diseases and Conditions Used in the Study
Table S3. Characteristics of Medicare Beneficiaries by Opioid and Benzodiazepine Trajectory Group
    Table S3a. (Opioid Use Only)
    Table S3b. (Benzodiazepine Use Only)
    Table S3c. (Opioid and benzodiazepine Use)
Table S4. Minimum and Maximum Standardized Mean Differences across Trajectory Group Comparisons
Table S5. Patterns of Opioid Use During 3-month Trajectory Measurement Period by Trajectory Group
Table S6. Patterns of Benzodiazepine Use During 3-month Trajectory Measurement Period by Trajectory Group
Table S7. Trajectories of Opioid and Benzodiazepine Use and 6-month Risk of Subsequent Injurious Falls among Medicare Beneficiaries
Table S8. E-values of Hazard Ratio Estimates for Injurious Falls among Medicare Beneficiaries
Figure S1. Sample Size Flowchart
Figure S2. Study Design Diagram
Figure S3. Trajectories of Opioid and Benzodiazepine Utilization Patterns and Risk of Injurious Falls: Sensitivity Analyses including Beneficiaries with Injurious Falls During the 6-month Trajectory Period

Table S1. Equivalency Conversion Table for Benzodiazepines

	Minimum diazepam 10 mg equivalent dose	Type (by half-life)
**Benzodiazepines**		
Alprazolam	0.5	Short-acting
Bromazepam	3	Intermediate-acting
Chlordiazepoxide	10	Long-acting
Clonazepam	0.5	Long-acting
Clorazepate	7.5	Long-acting
Diazepam	10	Long-acting
Estazolam	1	Intermediate-acting
Flurazepam	15	Long-acting
Lorazepam	1	Intermediate-acting
Nitrazepam	2.5	Long-acting
Oxazepam	15	Short-acting
Quazepam	15	Long-acting
Temazepam	10	Intermediate-acting
Triazolam	0.25	Short-acting

Table S2. ICD-9-CM and ICD-10-CM Codes of Diseases and Conditions Used in the Study

Diseases	ICD-9-CM	ICD-10-CM
Alcohol use disorders	303.x, 305.0x, 291	F10.1, F10.121, F10.14, F10.15, F10.180, F10.182, F10.2, F10.221, F10.231, F10.232, F10.24, F10.25, F10.26, F10.27, F10.280, F10.282
Anxiety disorders	293.84, 300.0X, 300.10, 300.2X, 300.3X, 300.89, 300.9X, 308.X, 309.81, 313.0, 313.1, 313.21, 313.22, 313.3X, 313.82, 313.83	F06.4, F40.x, F41.x, F42.x, F43.0, F43.1x, F44.9, F45.8, F48.8, F48.9, F93.8, F99, R45.7
Any cancers except for non-melanoma skin cancer	140.x, 141.x, 142.x, 143.x, 144.x, 145.x, 146.x, 147.x, 148.x, 149.x, 150.x, 151.x, 152.x, 153.x, 154.x, 155.x, 156.x, 157.x, 158.x, 159.x, 160.x, 161.x, 162.x, 163.x, 164.x, 165.x, 170.x, 171.x, 172.x, 174.x, 175.x, 176.x, 179.x, 180.x, 181.x, 182.x, 183.x, 184.x, 185.x, 186.x, 187.x, 188.x, 189.x, 190.x, 191.x, 192.x, 193.x, 194.x, 195.x, 196.x, 197.x, 198.x, 199.x, 200.x, 201.x, 202.x, 203.x, 204.x, 205.x, 206.x, 207.x, 208.x, 209.x, 210.x, 211.x, 212.x, 213.x, 214.x, 215.x, 216.x, 217.x, 218.x, 219.x, 220.x, 221.x, 222.x, 223.x, 224.x, 225.x, 226.x, 227.x, 228.x, 229.x, 230.x, 231.x, 232.x, 233.x, 234.x, 235.x, 236.x, 237.x, 238.x, 239.x	C00.x, C01.x, C02.x, C03.x, C04.x, C05.x, C06.x, C07.x, C08.x, C09.x, C10.x, C11.x, C12.x, C13.x, C14.x, C15.x, C16.x, C17.x, C18.x, C19.x, C20.x, C21.x, C22.x, C23.x, C24.x, C25.x, C26.x, C30.x, C31.x, C32.x, C33.x, C34.x, C37.x, C38.x, C39.x, C40.x, C41.x, C43.x, C4A.x, C45.x, C46.x, C47.x, C48.x, C49.x, C50.x, C51.x, C52.x, C53.x, C54.x, C55.x, C56.x, C57.x, C58.x, C60.x, C61.x, C62.x, C63.x, C64.x, C65.x, C66.x, C67.x, C68.x, C69.x, C70.x, C71.x, C72.x, C73.x, C74.x, C75.x, C76.x, C77.x, C78.x, C79.x, C80.x, C7A.x, C7B.x, C81.x, C82.x, C83.x, C84.x, C85.x, C86.x, C88.x, C90.x, C91.x, C92.x, C93.x, C94.x, C95.x, C96.x, D00.x, D01.x, D02.x, D03.x, D04.x, D05.x, D06.x, D07.x, D09.x, D10.x, D11.x, D12.x, D13.x, D14.x, D15.x, D16.x, D17.x, D18.x, D19.x, D20.x, D21.x, D22.x, D23.x, D24.x, D25.x, D26.x, D27.x, D28.x, D29.x, D30.x, D31.x, D32.x, D33.x, D34.x, D35.x, D36.x, D37.x, D38.x, D39.x, D40.x, D41.x, D42.x, D43.x, D44.x, D45.x, D46.x, D47.x, D48.x, D3A.x, D49.x
Injurious falls		
Falls	E880-E888	W00-W19
Fractures	800-804, 805, 806, 807.0x-807.1x, 808, 809, 810, 811, 812, 813, 814, 815, 816, 817, 818, 819, 820, 821, 822, 823, 824, 825, 826, 827, 828, 829, 831, 832, 833, 836	S02, S06, S12, S22, S32, S42, S43, S52, S53, S62, S63, S72, S81, S82, S92, T07, T148
	Procedure codes (only for outpatient settings): 27230-27248, 27193-27194, 27215-27228, 21800, 21805, 21810, 23500-23515, 23600-23630, 23665-23680, 24500-24587 plus splint, 24586, 24587, 24620, 24635, 24650-24685, 25500-25609, 25611, 25620, 25650-25652 plus cast or splint, 25622-25628 plus cast or splint, 26600, 26605, 26607, 26608, 26615, 26645, 26650, 26665, 26740, 26746 plus splint, 26720, 26725, 26727, 26735, 26740, 26742, 26746, 26750, 26755, 26756, 26765 plus splint, 27500-27514, 27530-27540, 27750-27759, 27780-27792, 27824-27828 plus cast or splint, 27520, 27524 plus splint, 27760-27769, 27808-27823, 28430-28445, 22305, 22310, 22315, 22318,	

	22319, 22325-22328, 22510-22515, 22520-22525, 27200, 27202, 72291, 72292, 76012, 76013, 77082, 77085, 77086, S2360-S2363	
Mood disorders	296.0x, 296.1x, 296.2x, 296.3x, 296.4x, 296.5x, 296.6x, 296.7x, 296.9x, 293.83, 296.80, 296.81, 296.82, 296.89, 311	F30.x, F32.x, F33.x, F31.x, F34.8, F39, F06.30, F31.9, F30.8, F32.8, F31.81, F32.9
Musculoskeletal system and connective tissues conditions	71y.x (y=0 to 9), 72y.x (y= 0 to 9), 73y.x (y=0 to 9),	M32.10, M33.20, M33.90, M34.0, M34.1, M34.9, M35.00, M35.01, M35.5, M35.8, M35.9, M11.20, M11.2x9 (x= 1 to 7), M11.28, M11.29, M11.80, M11.8x9 (x= 1 to 7), M11.88, M11.89, M11.9, M02.00, M02.20, M02.9, M12.80. M14.60. M14.80, M14.80. M36.2. M36.3, M36.4, M05.00, M05.10, M05.30, M05.60, M06.1, M06.4, M06.9. M08.00. M08.3, M08.40, M12.00, M15.x (x= 0 to 9), M16.10, M16.7, M16.9, M17.10, M17.5, M17.9, M18.9, M19.0x9 (x= 1 to 4, 7), M19.2x9 (x = 1 to 4, 7), M19.90, M19.91, M19.93, M12.10, M12.1x9 (x= 1 to 7), M12.18, M12.19, M12.50x, M12.8x, M12.9, M13.1x, M13.8x, M22.40, M23.009, M23.2x, M23.202, M23.205, M23.305, M23.3x9, M23.3x9 (x=1, 2, 3, 5, 6), M23.40, M23.50, M23.8x9, M24, M25, M45.9, M46.00, M46.1, M46.80, M46.90, M49.80, M47.10, M47.12. M47.14. M47.16, M47.812, M47.814, M47.817, M47.819. M48.10. M48.20, M48.30, M48.9, M46.45, M46.40, M46.47, M50.x0 (x=0 to 3, 8, 9), M51.0x, M51.2x, M51.3x, M51.4x, M51.8x, M51.9, M96.1, M43.6. M48.02. M53.0. M53.1, M53.82, M54.02. M54.12, M54.13, M54.2. M67.88, M43.27. M43.28, M43.8x9, M48.0x (x=0, 4, 6, 8). M53.2X7, M53.2X8, M53.3. M53.9, M54.08. M54.1x (x=4 to 7), M54.30, M54.5, M54.6, M54.89, M54.9, M35.3, M25.70, M25.729, M70.1x, M70.2x, M70.3x, M70.40, M70.5x. M70.6x, M70.7x, M75.0x, M75.10x. M75.2x, M75.3x, M75.4x, M75.5x, M75.8x, M76.x0 (x=1, 2, 4, 5, 6), M76.829, M76.899, M77.x0 (x=0 to 5, 8, 9), M67.47, M70.30, D48.1, M20.10, M21.61, M21.62, M65.00, M65.20, M65.30, M65.4, M65.80, M65.849, M65.879, M65.9, M66.10, M66.18, M66.2x9 (x =3 to 6). M66.339, M66.349. M66.369, M66.829, M66.879, M66.88, M66.9, M67.00, M67.4x (x=0 to 6), M67.50, M67.80. M67.88, M67.90, M70.039, M70.40, M71.00, M71.20, M71.x0 (x=3 to 5, 8, 9), M75.120, M24.20, M35.7, M60.009, M60.10, M60.20, M61.00, M61.10, M61.40, M61.59, M61.9, M62.00, M62.10, M62.3, M62.40, M62.50, M62.838, M62.84, M62.89, M62.9, M72.x (x =0, 1, 2, 4, 6), M54.10,

		M60.9, M70.98, M72.9. M79.0. M79.x (x= 1 to 5). M79.609. M79.7, M79.81. M79.89, M79.9, R25.2, R29.898, M46.20. M46.30, M86.1x, M86.2x, M86.6x, M86.9. M86.9, M89.6x, M90.8x, M88.9, M89.40. M89.70, M90.60, M90.80, M42.00, M42.10, M91.80, M92.x0 (x = 3 to 8), M93.003, M93.1, M93.20, M93.80, M93.90, M48.4, M48.50XA, M80, M81.x, M84, M85, M87, M89, M89.00, M89.30. M89.8X9, M94, M94.0, M94.8X9, S02.91XK, S02.91XK, S02.92XK. S12.000K, S12.001K, S12.100K, S12.101K, S12.200K, S12.201K, S12.300K, S12.301K, S12.400K, S12.401K, S12.500K. S12.501K, S12.600K S12.601K, S22.9XXK. S32.9XXK. S42.009K, S42.009P. S42.209K, S42.209P. S42.90XK, S42.90XP, S52.90XK, S52.90XM. S52.90XN, S52.90XP, S52.90XQ, S52.90XR, S62.90XK. S62.90XP. S72.90XK, S72.90XM, S72.90XN, S72.90XP, S72.90XQ, S72.90XR, S82.009P, S82.009Q, S82.009R, S82.90XK, S82.90XM, S82.90XN, S82.90XP, S82.90XQ, S82.90XR, S92.819K, S92.819P, S92.909K, S92.909P, S92.919K. S92.919P, S99.209K, S99.209P, S99.219K, S99.219P, S99.229K, S99.229P, S99.239K, 99.239P, S99.249K, S99.249P, S99.299K, S99.299P, M21.40, M20.10. M20.20, M20.30, M20.40, M20.5X9, M20.60, M20.009. M20.019, M20.029. M20.039. M20.099, M21.029, M21.059. M21.069, M21.129. M21.159, M21.169, M21.339, M21.5x, M21.6X9, M21.759, M21.769, M21.80, M21.839, M21.859, M21.869, M21.90, M21.939, M21.959, M21.969, M40.00, M40.10, M40.209, M40.299. M40.40. M40.50. M41.x0 (x=0, 2, 3, 4, 5, 8), M41.9, M43.8X9, M43.8x9, M96.2, M96.3, M96.4, M96.5, M43.00, M43.10, M89.38, M89.8x8, M95.x, M99.83, M99.84, M99
Non-opioid substance use disorders	304.1x, 304.2x, 304.3x, 304.4x, 304.6x, 304.8x, 304.9x, 305.2x, 305.3x, 305.4x, 305.6x, 305.7x, 305.8x, 305.9x, V11.3, V79.1, 303.x, 305.0x,	F10.1x, F10.220, F12.x, F13.1x, F13.2x, F13.90, F14.1x, F14.2x, F14.90, F15.1x, F15.2x, F15.90, F16.x, F18.10, F18.120, F18.20, F18.21, F18.90, F19.1x, F19.90, F55.x, Z65.8, Z13.89
Opioid use disorders	304.0x, 304.7x, 305.5x	F11.2x, F19.2x, F11.1x
Pain: abdominal pain	540.9, 553.1, 553.3, 564.1, 577.0, 533.90, 535.00, 550.90, 550.92, 553.2x (x=0,1,9), 590.80, 789.0x (x=0 to 7, 9), 541	K35.80, K35.89, K42.9, K44.9, K58.x (x=1, 2, 8, 9), K85.9x (x=0 to 2, 9) , K27.9, K29.00, K40.90, K40.20, K43.9, K43.2, K46.9, N12, R10.9, R10.11, R10.12, R10.31, R10.32, R10.33, R10.13, R10.84, R10.10, R10.2, R10.30, K37
Pain: fibromyalgia	729.1	M60.9, M79.1, M79.7

Pain: back pain	721.42, 721.5-721.91, 722.10, 722.2, 722.30, 722.32, 722.52, 722.6, 722.73, 722.80, 722.83, 722.90, 722.93, 724.00, 724.02, 724.09, 724.2, 724.3, 724.4, 724.5, 724.6, 724.8, 724.9, 737.1x, 737.20, 737.3x, 738.4, 739.3, 739.4, 756.10, 756.12-756.19, 805.4, 805.6, 805.8, 846.0-846.9, 307.89, 996.4x	F45.42, M40.00, M40.209, M40.299, M40.40, M41.00, M41.20, M41.30, M41.80, M41.9, M43.00, M43.10, M43.27, M43.28, M43.8X9, M46.40, M46.47, M47.10, M47.16, M47.819, M48.00, M48.061, M48.08, M48.10, M48.20, M48.30, M48.9, M51.06, M51.26, M51.27, M51.34, M51.35, M51.36, M51.37, M51.46, M51.47, M51.86, M51.87, M51.9, M53.2X7, M53.3, M53.9, M54.08, M54.14, M54.15, M54.16, M54.17, M54.30, M54.5, M54.89, M54.9, M96.1, M96.2, M96.3, M96.5, M97.9XXA, M99.03, M99.04, S12.9XXA, S22.009A, S32.009A, S32.10XA, S32.2XXA, S33.6XXA, S33.8XXA, S33.9XXA, T84.019A, T84.029A, T84.039A, T84.059A, T84.069A, T84.099A, T84.119A, T84.129A, T84.199A, T84.498A, Q76.0, Q76.1, Q76.2, Q76.419, Q76.49
Pain: chest	413.9, 786.5x (x=0 to 2, 9)	I20.8, I20.9, R07.9, R07.2, R07.1, R07.8x (x=1,2,9)
Pain: headache/ migraine	346.xx, 784.0x, 307.81,	G43.019, G43.109, G43.119, G43.711, 43.809, G43.819, G43.909, G43.919, G43.yx (y=A to D), G44.1, R51, G44.209
Pain: neck pain	723.xx, 721.0x, 721.1x, 722.0x, 722.4x, 839.0x, 839.1x, 847.0x, 722.x1 (x=3,7,8,9)	M48.02, M53.0, M53.1, M54.2, M47.812, M47.12, M50.20, M50.30, M43.6, M53.82, M54.02, M54.12, M54.13, M67.88, S11.90XA, S11.90XA,S13.1x1A (x=0 to 8), S13.4XXA, S13.8XXA, M51.44, M51.45, M50.00, M96.1, M50.80, M50.90
Pain: osteoarthritis	715.x	M15.x, M16.x, M17.x, M18.x, M19.x
Pain: rheumatoid arthritis	714.x	M05.x, M06.x, M08.yx (y=0,2,3,8,9), M12.0
Pain: temporomandibular disorder pain	524.6x (x=0 to 3, 9)	M26.60x (x=1 to 3, 9), M26.69, M26.61x (x=1 to 3, 9), M26.62x (x=1 to 3, 9), M26.63x (x=1 to 3, 9)
Pain: pelvic (e.g., menstrual/ genital reproductive) pain	625.x (x=3,8,9), 626.6, 627.1, 627.2	N94.6, N94.89, R10.2, N92.1, N95.0, N95.1
Other pain conditions	996.4x, 72y.xx (y=5 to 9), 382.9, 522.4, 522.5, 525.9, 565.1, 703.0, 706.2, 848.x (x=3,8,9), 379.91, 380.22, 380.23, 381.81, 388.70, 569.42, 604.90, 611.71, 611.79, 719.30, 786.52, 873.63, 470, 338.0, G89.2, 338.4	M97.9XXA, T84.0x9A (x=1 to 3, 5, 6, 9), T84.099A, T84.1x9A (x=1,2,9), T84.498A, M35.3, M25.70, M25.729, M70.yx (y=1 to 3, 5 to 7), M70.40, M75.0x, M75.10x, M75.yx (y=2 to 5,8), M76.x0 (x=1,2,4 to 6), M76.829, M76.899, M77.x0 (x=0 to 5), M77.8, M77.9, M67.47, M70.30, D48.1, M20.10, M21.61, M21.62, M65.00, M65.20, M65.30, M65.4, M65.80, M65.80, M65.849, M65.879, M65.9, M66.10, M66.18, M66.2x9 (x=3 to 6), M66.3x9 (x=4 to 6), M66.349, M66.369, M66.829,

		M66.879, M66.88, M66.9, M67.00, M67.4x (x=0 to 6), M67.50, M67.80, M67.88, M67.90, M70.039, M70.40, M71.x0 (x=0,2 to 5,8), M71.9, M75.120, M24.20, M35.7, M60.009, M60.10, M60.20, M61.x0 (x=0,1,4), M61.59, M61.9, M62.x0 (x=0,1,4,5), M62.3, M62.838, M62.84, M62.89, M62.9, M72.x (x=0 to 2,4,6), M54.10, M60.9, M70.98, M72.9, M79.x (x=0 to 5), M79.609, M79.7, M79.81, M79.89, M79.9, R25.2, R29.898, H66.90, K04.4, K04.7, K08.9, K60.x (x=3 to 5), L60.0, L72.3, S23.41XA, S03.9XXA, S29.019A, S39.011A, T14.90XA, H57.13, H60.5x9 (x=0 to 5, 9), H60.60, H60.8X1, H60.90, H69.80, H92.09, K62.89, N45.x (x=1 to 3), N64.4, N64.5x (x=1 to 3, 9), M12.30, M12.40, R07.1, R07.81, S02.5XXA, S02.5XXB, J34.2, G89.x (x=0,2,4)
Sleep disorders	291.82, 292.85, 307.4, 307.41, 307.42, 307.45, 307.46, 307.47, 307.48, 307.49, 327.11, 327.12, 327.2, 327.21, 327.23, 327.24, 327.26, 327.27, 327.29, 327.3, 327.31, 327.32, 327.33, 327.34, 327.35, 327.36, 327.37, 327.39, 327.42, 327.43, 327.52, 327.53, 327.59, 327.8, 333.94, 347.0, 347.00, 347.01, 347.10, 347.11, 780.5, 780.51, 780.53, 780.55, 780.56, 780.57, 780.58, 780.59, V69.4	F51, F51.0, F51.1, F51.11, F51.13, F51.19, F51.2, F51.3, F51.4, F51.5, F51.8, F51.9, G25.8, G47, G47.0, G47.1, G47.10, G47.11, G47.12, G47.13, G47.14, G47.19, G47.2, G47.30, G47.31, G47.33, G47.34, G47.37, G47.39, G47.4, G47.411, G47.419, G47.421, G47.429, G47.8, G47.9, R06.81

Table S3a. Characteristics of Medicare Beneficiaries by Opioid and Benzodiazepine Trajectory Group (Opioid Use Only)

Trajectory groups	Opioid use only				
	A: n=279,263 (44.9%)	B: n=93,703 (15.1%)	C: n=47,851 (7.7%)	D: n=24,952 (4.0%)	E: n=14,225 (2.3%)
Age ≥65 years, %	85.5	85.1	87.3	82.8	78.0
Female, %	53.8	53.3	64.3	62.9	52.9
Race/ethnicity group, %					
White	82.2	83.7	76.3	77.2	83.4
Black	9.5	9.3	12.5	13.1	10.2
Others	8.2	6.9	11.1	9.7	6.4
Disability status, %	19.8	21.1	21.6	27.4	30.9
LIS/Dual eligibility, %					
No LIS/dual eligibility	75.5	76.1	66.6	58.9	69.6
LIS or dual eligibility	5.0	4.8	5.4	7.0	7.9
LIS and dual eligibility	19.5	19.1	27.9	34.1	22.5
Metropolitan residence	81.6	80.0	81.6	79.2	79.4
Elixhauser Comorbidity Index, mean (SD)	3.1 (2.6)	3.6 (2.7)	3.8 (2.7)	4.1 (2.9)	3.6 (2.8)
Opioid use disorder, %	0.2	0.4	0.3	0.5	1.5
Alcohol use disorders, %	1.0	1.2	0.9	1.4	1.8
Other SUD, %	0.6	0.7	0.5	0.7	1.2
Anxiety disorders, %	6.7	8.3	7.3	9.3	9.7
Mood disorders, %	9.5	11.6	10.8	15.0	14.3
Sleep disorders, %	13.2	16.8	14.4	16.6	19.5
Musculoskeletal conditions, %	41.3	58.8	59.6	63.3	71.4
Pain conditions, %					
Osteoarthritis	32.4	46.3	44.8	47.3	53.1
Low back pain	17.5	25.2	31.7	36.0	36.6
Neck pain	6.6	9.0	10.7	11.3	12.5
Chest pain	11.5	13.5	12.9	13.5	12.1
Abdominal pain	19.3	20.2	15.5	16.3	14.7
Rheumatoid arthritis	2.1	2.9	4.5	4.9	3.9
Pelvic pain	3.1	3.1	2.7	2.5	2.5
Headache/migraine	4.6	5.1	5.5	5.8	5.6
TMJ	0.2	0.2	0.2	0.2	0.1
Others	19.9	25.3	25.0	26.3	27.2
Any hospitalization, %	11.0	28.0	10.2	12.1	29.2
ED visits, %					
0	88.5	87.5	88.2	85.6	87.4
1	10.1	10.7	10.2	12.1	10.7
≥2	1.4	1.8	1.6	2.3	1.9
Outpatient visits, %					
0	38.3	29.1	40.3	37.6	32.0
1	23.6	23.3	22.7	22.3	23.3
2-5	32.9	39.9	32.3	34.2	38.0
>5	5.2	7.7	4.7	5.9	6.7

No. antidepressants	0.7 (1.9)	0.7 (1.9)	0.8 (2.1)	1.1 (2.5)	0.9 (2.2)
No. antipsychotics	0.2 (1.4)	0.2 (1.2)	0.2 (1.3)	0.3 (1.6)	0.2 (1.1)
No. gabapentinoids	0.2 (0.9)	0.3 (1.1)	0.4 (1.2)	0.6 (1.6)	0.4 (1.3)
No. muscle relaxants	0.1 (0.5)	0.1 (0.6)	0.1 (0.6)	0.2 (0.9)	0.2 (0.7)
No. naltrexone	0.0 (0.1)	0.0 (0.1)	0.0 (0.1)	0.0 (0.1)	0.0 (0.1)
Polypharmacy, %	86.1	88.2	91.7	91.7	85.6

Abbreviations: ED, emergency department; LIS, low-income subsidy; No., number of; SD, standard deviation; SUD: substance use disorder; TMJ: temporomandibular disorder pain.

Table S3b. Characteristics of Medicare Beneficiaries by Opioid and Benzodiazepine Trajectory Group (Benzodiazepine Use Only)

Trajectory groups	Benzodiazepine use only			
	F: n=71,715 (11.5%)	G: n=28,109 (4.5%)	H: n=19,230 (3.1%)	I: n=13,013 (2.1%)
Age ≥65 years, %	85.7	82.0	83.9	69.3
Female, %	70.9	66.3	59.5	56.8
Race/ethnicity group, %				
White	86.8	84.4	84.3	82.9
Black	5.7	6.2	5.9	6.7
Others	7.5	9.4	9.7	10.4
Disability status, %	19.5	24.3	22.9	38.1
LIS/Dual eligibility, %				
No LIS/dual eligibility	74.8	68.1	65.9	56.3
LIS or dual eligibility	4.2	5.1	5.2	7.8
LIS and dual eligibility	21.1	26.9	29.0	35.9
Metropolitan residence	85.5	84.4	80.6	83.8
Elixhauser Comorbidity Index, mean (SD)	3.1 (2.5)	3.3 (2.7)	3.4 (2.6)	3.5 (2.8)
Opioid use disorder, %	0.3	0.5	0.3	1.6
Alcohol use disorders, %	1.1	1.9	1.2	2.8
Other SUD, %	0.7	1.5	0.9	3.2
Anxiety disorders, %	20.8	26.1	19.2	30.7
Mood disorders, %	14.6	20.2	16.9	29.4
Sleep disorders, %	14.3	18.7	16.3	24.0
Musculoskeletal conditions, %	43.1	40.3	36.6	36.3
Pain conditions, %				
Osteoarthritis	34.0	31.2	28.9	27.1
Low back pain	17.7	16.3	14.2	15.9
Neck pain	7.8	7.0	5.8	6.5
Chest pain	12.2	14.0	11.2	12.1
Abdominal pain	13.2	13.5	11.5	12.5
Rheumatoid arthritis	2.0	2.2	1.9	2.0
Pelvic pain	3.6	3.1	2.1	2.5
Headache/migraine	6.2	6.4	4.6	6.4
TMJ	0.3	0.3	0.2	0.2
Others	19.7	18.0	16.0	17.0
Any hospitalization, %	7.8	11.5	9.0	14.8
ED visits, %				
0	89.4	85.5	88.3	83.7
1	9.1	12.2	10.0	13.4
≥2	1.5	2.3	1.7	2.9
Outpatient visits, %				
0	42.7	45.1	43.9	46.8
1	23.6	23.2	23.2	23.4
2-5	29.8	28.2	29.4	26.5
>5	3.9	3.6	3.5	3.3

No. antidepressants	1.0 (2.4)	1.3 (2.6)	1.2 (2.7)	1.8 (3.2)
No. antipsychotics	0.4 (2.1)	0.7 (2.8)	0.6 (2.5)	1.4 (3.8)
No. gabapentinoids	0.2 (0.9)	0.2 (1.0)	0.2 (1.0)	0.3 (1.2)
No. muscle relaxants	0.1 (0.6)	0.1 (0.6)	0.1 (0.6)	0.1 (0.7)
No. naltrexone	0.0 (0.1)	0.0 (0.2)	0.0 (0.1)	0.0 (0.2)
Polypharmacy, %	87.7	88.5	90.0	87.0

Abbreviations: ED, emergency department; LIS, low-income subsidy; No., number of; SD, standard deviation; SUD: substance use disorder; TMJ: temporomandibular disorder pain.

Table S3c. Characteristics of Medicare Beneficiaries by Opioid and Benzodiazepine Trajectory Group (Opioid and Benzodiazepine Use)

Trajectory groups	Combination use			
	J: n=17,750 (2.9%)	K: n=5,601 (0.9%)	L: n=3,729 (0.6%)	M: n=3,447 (0.6%)
Age ≥65 years, %	85.7	81.6	79.5	64.5
Female, %	63.8	65.5	63.0	57.3
Race/ethnicity group, %				
White	86.8	84.1	85.3	84.4
Black	6.3	7.7	6.5	8.2
Others	6.9	8.2	8.2	7.5
Disability status, %	19.6	25.1	28.2	43.9
LIS/Dual eligibility, %				
No LIS/dual eligibility	78.3	70.2	63.4	58.6
LIS or dual eligibility	4.0	4.9	7.5	11.9
LIS and dual eligibility	17.7	24.9	29.1	29.4
Metropolitan residence	84.3	82.8	80.2	80.1
Elixhauser Comorbidity Index, mean (SD)	3.2 (2.7)	3.8 (2.9)	4.0 (3.1)	3.7 (3.1)
Opioid use disorder, %	0.4	0.5	0.6	2.6
Alcohol use disorders, %	1.5	1.4	1.3	3.5
Other SUD, %	0.8	1.2	1.3	2.4
Anxiety disorders, %	14.7	17.8	24.6	25.7
Mood disorders, %	13.0	17.5	20.6	24.3
Sleep disorders, %	16.6	19.2	21.9	21.7
Musculoskeletal conditions, %	51.9	54.4	48.7	58.5
Pain conditions, %				
Osteoarthritis	40.2	42.2	37.6	41.9
Low back pain	25.7	27.2	24.1	36.9
Neck pain	10.6	10.5	9.3	15.1
Chest pain	13.8	15.7	17.4	15.5
Abdominal pain	16.5	21.8	19.4	20.1
Rheumatoid arthritis	2.7	3.1	2.7	3.3
Pelvic pain	4.1	4.3	3.6	4.4
Headache/migraine	7.0	7.9	7.9	7.9
TMJ	0.3	0.4		
Others	24.9	26.4	23.4	26.3
Any hospitalization, %	12.9	16.3	17.9	20.6
ED visits, %				
0	87.8	83.0	81.2	81.5
1	10.3	14.0	15.7	15.1
≥2	2.0	3.0	3.1	3.4
Outpatient visits, %				
0	38.4	34.2	37.0	37.8
1	22.9	23.1	23.0	22.2
2-5	33.3	35.8	34.1	33.8

>5	5.4	6.9	6.0	6.2
No. antidepressants	0.9 (2.1)	1.2 (2.4)	1.2 (2.5)	1.2 (2.6)
No. antipsychotics	0.2 (1.2)	0.4 (2.0)	0.4 (2.0)	0.5 (3.6)
No. gabapentinoids	0.2 (0.9)	0.3 (1.1)	0.3 (1.2)	0.4 (1.4)
No. muscle relaxants	0.1 (0.6)	0.1 (0.7)	0.1 (0.6)	0.2 (0.8)
No. naltrexone	0.0 (0.1)	0.0 (0.1)	0.0 (0.0)	0.0 (0.1)
Polypharmacy, %	87.9	90.9	88.5	78.0

Abbreviations: ED, emergency department; LIS, low-income subsidy; No., number of; SD, standard deviation; SUD: substance use disorder; TMJ: temporomandibular disorder pain.

Table S4. Minimum and Maximum Standardized Mean Differences across Trajectory Group Comparisons

	Min*		Max*	
	**Unwt.**	**Wt.**	**Unwt.**	**Wt.**
Age ≥65 years	0.001	<0.001	0.554	0.034
Female	0.003	0.001	0.379	0.050
Race/ethnicity group				
White	<0.001	<0.001	0.273	0.059
Black	0.004	<0.001	0.255	0.061
Others	<0.001	<0.001	0.168	0.039
Disability status	0.002	<0.001	0.543	0.030
LIS/Dual eligibility				
No LIS/no dual eligibility	0.005	<0.001	0.482	0.051
LIS or dual eligibility	0.003	<0.001	0.296	0.039
LIS and dual eligibility	0.003	<0.001	0.420	0.046
Metropolitan residence	<0.001	0.001	0.166	0.046
Elixhauser Comorbidity Index	0.008	<0.001	0.370	0.033
Opioid use disorder	0.001	<0.001	0.203	0.028
Alcohol use disorders	0.002	0.001	0.176	0.049
Other SUD	0.001	<0.001	0.198	0.044
Anxiety disorders	0.010	<0.001	0.645	0.060
Mood disorders	0.010	0.001	0.519	0.041
Sleep disorders	0.001	<0.001	0.279	0.029
Musculoskeletal conditions	0.005	0.001	0.752	0.069
Pain conditions				
Osteoarthritis	0.004	<0.001	0.551	0.062
Low back pain	0.006	<0.001	0.538	0.056
Neck pain	0.003	<0.001	0.306	0.016
Chest pain	<0.001	<0.001	0.177	0.034
Abdominal pain	0.002	<0.001	0.278	0.043
Rheumatoid arthritis	0.003	<0.001	0.169	0.032
Pelvic pain	<0.001	<0.001	0.127	0.043
Headache/migraine	<0.001	<0.001	0.140	0.027
TMJ	<0.001	<0.001	0.059	0.062
Others	0.001	<0.001	0.275	0.028
Any hospitalization	0.015	0.001	0.573	0.130
ED visits				
0	0.002	<0.001	0.232	0.027
1	0.001	<0.001	0.202	0.024
≥2	0.001	<0.001	0.130	0.014
Outpatient visits				
0	0.003	<0.001	0.370	0.065
1	<0.001	<0.001	0.034	0.034
2-5	0.003	0.001	0.287	0.057
>5	0.002	<0.001	0.191	0.046
No. antidepressants	0.002	<0.001	0.446	0.046
No. antipsychotics	0.001	<0.001	0.439	0.058

No. gabapentinoids	0.006	<0.001	0.303	0.034
No. muscle relaxants	0.007	<0.001	0.194	0.042
No. naltrexone	0.001	<0.001	0.058	0.021
Polypharmacy	<0.001	<0.001	0.390	0.025

Abbreviations: ASMD; absolute standardized mean difference; ED, emergency department; LIS, low-income subsidy; No., number of; SD, standard deviation; SUD: substance use disorder; TMJ: temporomandibular disorder pain; Unwt, unweighted; Wt, weighted

* Represents the minimum and maximum ASMD across the 78 ASMDs from group comparisons (the number of 2-combinations from the given 13 trajectories: $C_2^{13} = 78$; i.e., group A vs B, group A vs C, group A vs D).

Table S5. Patterns of Opioid Use During 3-month Trajectory Measurement Period by Trajectory Group

OPI use patterns*	Overall	A	B	C	D	E	F	G	H	I	J	K	L	M
No. beneficiaries	622588	279263	93703	47851	24952	14225	71715	28109	19230	13013	17750	5601	3729	3447
% of the overall cohort	100.0	44.9	15.1	7.7	4.0	2.3	11.5	4.5	3.1	2.1	2.9	0.9	0.6	0.6
**Type of OPIs, %**														
SAO Schedule II only	59.5	66.6	30.3	64.1	24.4	49.5	0	0	0	0	65.3	55.1	54.2	52.3
SAO Schedule III only	10.5	14.5	7.9	3.6	4.7	0.9	0	0	0	0	7.9	9.3	9.6	3.8
SAO Schedule IV only	21.6	17.9	54.6	11.6	48.1	6.2	0	0	0	0	18.3	22.3	26.7	13.8
LAO Schedule II only	0.1	0	0	0.2	0.1	2.5	0	0	0	0	0.1	0.1	0.3	1.3
SAO mixed Schedules	7.5	0.9	6.9	19.4	21.2	30.2	0	0	0	0	7.8	12.8	8.5	18.7
SAO and LAO mixed use	0.5	0	0.1	0.8	0.5	8.5	0	0	0	0	0.3	0.1	0.5	8.8
Others	0.3	0	0.2	0.4	1.1	2.1	0	0	0	0	0.3	0.3	0.3	1.2
**OPI medication (top 10), %**														
Hydrocodone SAO	45.8	49.3	27.3	46.9	33.0	51.7	0	0	0	0	47.2	46.3	41.5	47.4
Oxycodone SAO	21.0	15.7	6.0	40.8	10.1	52.9	0	0	0	0	26.0	21.0	19.1	40.1
Codeine SAO	12.9	15.0	10.4	9.8	11.4	7.5	0	0	0	0	10.3	13.4	12.2	8.7
Diphenoxylate	2.2	2.1	3.9	1.2	3.9	0.6	0	0	0	0	1.5	3.1	3.0	1.7
Hydromorphone SAO	0.9	0.4	0.2	2.3	0.4	4.9	0	0	0	0	1.4	1.2	1.1	3.8
Fentanyl LAO	0.2	0	0	0.4	0.1	3.6	0	0	0	0	0.1	0	0.2	3.4
Morphine LAO	0.2	0	0	0.4	0.1	1.4	0	0	0	0	0.6	0.3	0.4	2.3
Morphine SAO	0.2	0	0	0.1	0.1	3.5	0	0	0	0	0.1	0	0.3	2.8
Oxycodone LAO	0.2	0	0	0.3	0	2.2	0	0	0	0	0.1	0.1	0.1	1.9
Buprenorphine	0	0	0.1	0	0.1	0.3	0	0	0	0	0	0	0.1	0.3
**Other treatment patterns**														
First OPI prescription's days supplied, mean (SD)	8.2 (9.5)	4.2 (2.3)	21.4 (9.6)	7.7 (5.4)	24.2 (20.2)	15.6 (12.8)	0 (0)	0 (0)	0 (0)	0 (0)	6.9 (7.5)	9.0 (10.1)	11.6 (13.1)	15.9 (13.6)
Mean OPI prescription's days supplied, (SD)	8.4 (9.4)	4.2 (2.3)	21.6 (9.0)	7.9 (5.1)	26.2 (18.3)	16.9 (10.9)	0 (0)	0 (0)	0 (0)	0 (0)	7.2 (7.4)	9.2 (9.8)	12.1 (12.9)	16.9 (12.4)
Mean daily OPI MME, (SD)	2.7 (5.1)	1.3 (0.6)	3.5 (1.7)	5.1 (3.0)	8.3 (4.0)	23.7 (15.2)	0 (0)	0 (0)	0 (0)	0 (0)	2.8 (2.9)	3.1 (2.7)	3.9 (4.3)	18.6 (19.3)
Mean no. OPI fills, (SD)	1.4 (0.9)	1.0 (0.2)	1.3 (0.6)	1.8 (0.8)	2.9 (1.3)	3.9 (1.9)	0 (0)	0 (0)	0 (0)	0 (0)	1.4 (0.7)	1.6 (0.9)	1.5 (1.0)	3.1 (2.2)

Abbreviations: LAO, long-acting opioids; No, number of; OPI, opioid; SAO, short-acting opioids

* To facilitate the labeling of opioid and benzodiazepine dose levels for each trajectory, we defined opioid dosage use as: very-low- (SDD <25 MME), low- (25–50 MME), moderate- (51–90 MME), high- (91–150 MME), and very-high-dose (>150 MME). Similarly, we defined BZD dosage use as very-low- (<10 DME), low- (10–20 DME), moderate- (21–40 DME), high- (41–60 DME), and very-high-dose (>60 DME). Trajectory groups: **A**: Very-low OPI-only (early discontinuation); **B**: Low OPI-only (rapid decline); **C**: Very-low OPI-only (late discontinuation); **D**: Low OPI-only (gradual decline); **E**: Moderate OPI-only (rapid decline); **F**: Very-low BZD-only (late discontinuation); **G**: Low BZD-only (rapid decline); **H**: Low BZD-only (stable); **I**: Moderate BZD-only (gradual decline); **J**: Very-low OPI (rapid decline) / Very-low BZD (late discontinuation); **K**: Very-low OPI (rapid decline)/ Very-low BZD (increasing); **L**: Very-low OPI (stable)/Low BZD (stable); **M**: Low OPI (gradual decline)/ Low BZD (gradual decline).

Table S6. Patterns of Benzodiazepine Use During 3-month Trajectory Measurement Period by Trajectory Group

BZD use patterns*	Overall	A	B	C	D	E	F	G	H	I	J	K	L	M
No. beneficiaries	622588	279263	93703	47851	24952	14225	71715	28109	19230	13013	17750	5601	3729	3447
% of the overall cohort	100	44.9	15.1	7.7	4.0	2.3	11.5	4.5	3.1	2.1	2.9	0.9	0.6	0.6
**Type of BZD use, %**														
SA only	34.3	0	0	0	0	0	29.1	24.7	58.2	33.0	44.5	35.4	38.5	35.8
IA only	33.3	0	0	0	0	0	39.4	35.1	21.3	23.8	28.1	32.1	26.7	31.8
LA only	29.5	0	0	0	0	0	30.9	35.1	18.3	33.8	25.6	28.6	27.9	23.8
SA and IA mixed	1.0	0	0	0	0	0	0.2	1.6	0.7	3.8	0.8	1.3	2.2	3.3
SA and LA mixed	0.9	0	0	0	0	0	0.2	2.0	0.6	2.7	0.4	1.0	2.5	2.5
LA and IA mixed	0.8	0	0	0	0	0	0.2	1.4	0.6	2.4	0.7	1.5	2.0	2.4
SA, IA and LA mixed	0.1	0	0	0	0	0	0	0.1	0	0.4	0	0.1	0.2	0.4
Missing	0.1	0	0	0	0	0	0	0	0.3	0.1	0	0.1	0.1	0
**BZD ingredients, %**														
Alprazolam	34.6	0	0	0	0	0	39.3	38.3	22.2	29.1	27.6	34.2	30.8	36.9
Lorazepam	25.9	0	0	0	0	0	30.0	26.1	18.4	19.5	23.8	23.9	23.0	19.5
Diazepam	17.2	0	0	0	0	0	20.7	7.5	5.6	3.0	40.9	21.7	10.5	21.3
Clonazepam	10.7	0	0	0	0	0	6.0	16.0	10.7	31.6	3.9	10.2	14.2	19.5
Chlordiazepoxide	8.4	0	0	0	0	0	2.8	4.5	43.4	5.7	1.2	6.4	18.9	2.1
Temazepam	5.8	0	0	0	0	0	1.3	13.0	1.3	22.8	3.0	7.3	10.2	11.4
Triazolam	0.5	0	0	0	0	0	0.5	0.2	0.3	0.1	1.5	0.5	0.5	0.2
Clobazam	0.1	0	0	0	0	0	0	0.1	0.3	0.2	0	0.1	0.1	0
Oxazepam	0.1	0	0	0	0	0	0.1	0.1	0.1	0.1	0.1	0	0.1	0.1
Flurazepam	0	0	0	0	0	0	0	0.1	0	0.1	0	0	0.1	0
Estazolam	0	0	0	0	0	0	0	0	0	0	0	0.1	0.1	0.1
**Other treatment patterns**														
First BZD prescription's days supplied, mean (SD)	23.7 (22.7)	0 (0)	0 (0)	0 (0)	0 (0)	0 (0)	14.6 (11.7)	25.0 (12.5)	56.3 (31.2)	37.8 (25.8)	9.7 (10.7)	21.3 (20.5)	42.0 (28.2)	23.7 (18.0)
Mean BZD prescription's days supplied, (SD)	24.2 (22.5)	0 (0)	0 (0)	0 (0)	0 (0)	0 (0)	14.6 (11.7)	25.9 (11.8)	57.6 (29.8)	39.3 (24.3)	9.8 (10.6)	21.9 (20.1)	43.5 (26.9)	24.6 (17.3)

Mean daily BZD DME, (SD)	1.3 (4.2)	0 (0)	0 (0)	0 (0)	0 (0)	0 (0)	1.5 (1.1)	5.9 (2.9)	7.9 (2.5)	21.7 (10.4)	1.4 (1.7)	2.5 (2.4)	8.7 (4.3)	14.2 (14.4)
Mean no. BZD fills, (SD)	1.5 (1.0)	0 (0)	0 (0)	0 (0)	0 (0)	0 (0)	1.1 (0.3)	1.7 (0.8)	2.3 (1.3)	2.9 (1.6)	1.1 (0.3)	1.3 (0.6)	2.4 (1.2)	2.1 (1.3)

Abbreviations: BZD, benzodiazepine; IA, intermediate-acting; LA, long-acting; No, number of; SA, short-acting;

* To facilitate the labeling of opioid and benzodiazepine dose levels for each trajectory, we defined BZD dosage use as very-low- (<10 DME), low- (10–20 DME), moderate- (21–40 DME), high- (41–60 DME), and very-high-dose (>60 DME). Trajectory groups: **A**: Very-low OPI-only (early discontinuation); **B**: Low OPI-only (rapid decline); **C**: Very-low OPI-only (late discontinuation); **D**: Low OPI-only (gradual decline); **E**: Moderate OPI-only (rapid decline); **F**: Very-low BZD-only (late discontinuation); **G**: Low BZD-only (rapid decline); **H**: Low BZD-only (stable); **I**: Moderate BZD-only (gradual decline); **J**: Very-low OPI (rapid decline) / Very-low BZD (late discontinuation); **K**: Very-low OPI (rapid decline)/ Very-low BZD (increasing); **L**: Very-low OPI (stable)/Low BZD (stable); **M**: Low OPI (gradual decline)/ Low BZD (gradual decline).

Table S7. Trajectories of Opioid and Benzodiazepine Use and 6-month Risk of Subsequent Injurious Falls among Medicare Beneficiaries

	Injurious Falls (n=5,167)			
	N (crude rate*)	Days of follow-up, median (IQR)	HR (95%CI)	
Trajectory Groups§			Unadjusted	Adjusted†
A. Very-low OPI-only (early discontinuation)	1,930 (11.6)	83 (88.0)	Ref	Ref
B. Low OPI-only (rapid decline)	641 (11.4)	91 (83.0)	0.99 (0.91, 1.08)	1.00 (0.92, 1.09)
C. Very-low OPI-only (late discontinuation)	583 (20.4)	69 (95.0)	1.77 (1.61, 1.94)	1.54 (1.40, 1.69)
D. Low OPI-only (gradual decline)	354 (23.8)	71 (75.0)	2.06 (1.84, 2.31)	1.93 (1.72, 2.16)
E. Moderate OPI-only (rapid decline)	185 (21.8)	66 (83.0)	1.89 (1.63, 2.20)	2.22 (1.93, 2.55)
F. Very-low BZD-only (late discontinuation)	521 (12.2)	87 (82.0)	1.05 (0.95, 1.16)	0.95 (0.86, 1.04)
G. Low BZD-only (rapid decline)	237 (14.1)	90 (85.0)	1.22 (1.07, 1.40)	1.12 (0.98, 1.29)
H. Low BZD-only (stable)	252 (22.0)	76 (93.5)	1.90 (1.67, 2.17)	1.82 (1.59, 2.07)
I. Moderate BZD-only (gradual decline)	143 (18.4)	101 (62.0)	1.59 (1.34, 1.89)	1.31 (1.09, 1.58)
J. Very-low OPI (rapid decline)/Very-low BZD (late discontinuation)	150 (14.2)	92 (68.0)	1.22 (1.04, 1.44)	1.08 (0.91, 1.28)
K. Very-low OPI (rapid decline)/Very-low BZD (increasing)	43 (12.8)	109 (73.0)	1.11 (0.82, 1.50)	0.99 (0.73, 1.36)
L. Very-low OPI (stable)/Low BZD (stable)	80 (36.2)	84 (74.5)	3.13 (2.50, 3.91)	2.64 (2.08, 3.36)
M. Low OPI (gradual decline)/Low BZD (gradual decline)	48 (23.4)	79 (68.0)	2.02 (1.52, 2.69)	1.72 (1.26, 2.35)

Abbreviations: CI, confidence interval; BZD, benzodiazepines; HR, hazard ratio; OPI, opioid; OPI-BZD, concurrent opioid and benzodiazepine use

§ To facilitate the labeling of opioid and benzodiazepine dose levels for each trajectory, we defined opioid dosage use as: very-low- (SDD <25 MME), low- (25–50 MME), moderate- (51–90 MME), high- (91–150 MME), and very-high-dose (>150 MME). Similarly, we defined BZD dosage use as very-low- (<10 DME), low- (10–20 DME), moderate- (21–40 DME), high- (41–60 DME), and very-high-dose (>60 DME).

* The unit for crude rates is per 10,000 person-months.

† We excluded 11 beneficiaries with extreme IPTWs (>10) using trimming methods to increase validity of treatment effect estimates.

Table S8. E-values of Hazard Ratio Estimates for 3-month Injurious Falls among Medicare Beneficiaries

	Injurious Falls		
**Trajectory Groups§**	**Adjusted HRs (95% CI)**	**E-value for HR***	**E-value for lower 95% CI**
A. Very-low OPI-only (early discontinuation)	Ref	Ref	Ref
B. Low OPI-only (rapid decline)	0.92 (0.81, 1.03)	1.36	1.00
C. Very-low OPI-only (late discontinuation)	1.78 (1.58, 2.01)	3.48	3.00
D. Low OPI-only (gradual decline)	2.24 (1.93, 2.59)	4.17	3.52
E. Moderate OPI-only (rapid decline)	2.60 (2.18, 3.09)	4.07	3.25
F. Very-low BZD-only (late discontinuation)	0.93 (0.81, 1.07)	1.24	1.00
G. Low BZD-only (rapid decline)	1.02 (0.84, 1.24)	1.62	1.00
H. Low BZD-only (stable)	2.02 (1.70, 2.40)	3.54	2.87
I. Moderate BZD-only (gradual decline)	1.03 (0.77, 1.36)	1.69	1.00
J. Very-low OPI (rapid decline) / Very-low BZD (late discontinuation)	0.99 (0.78, 1.26)	1.46	1.00
K. Very-low OPI (rapid decline)/ Very-low BZD (increasing)	0.59 (0.34, 1.02)	2.35	1.00
L. Very-low OPI (stable)/Low BZD (stable)	2.73 (1.98, 3.76)	6.42	4.66
M. Low OPI (gradual decline)/ Low BZD (gradual decline)	1.96 (1.32, 2.91)	3.64	2.24

Abbreviations: CI, confidence interval; BZD, benzodiazepines; HR, hazard ratio; OPI, opioid

§ To facilitate the labeling of opioid and benzodiazepine dose levels for each trajectory, we defined opioid dosage use as: very-low (SDD <25 MME), low (25–50 MME), and moderate (51–90 MME). Similarly, we defined BZD dosage use as very-low (<10 DME), low (10–20 DME), and moderate (21–40 DME).

* E-value is defined as the minimum strength of an association that an unmeasured confounder would need to have with the treatment and outcome to fully account for a specific treatment-outcome association, conditioned on the measured covariates.

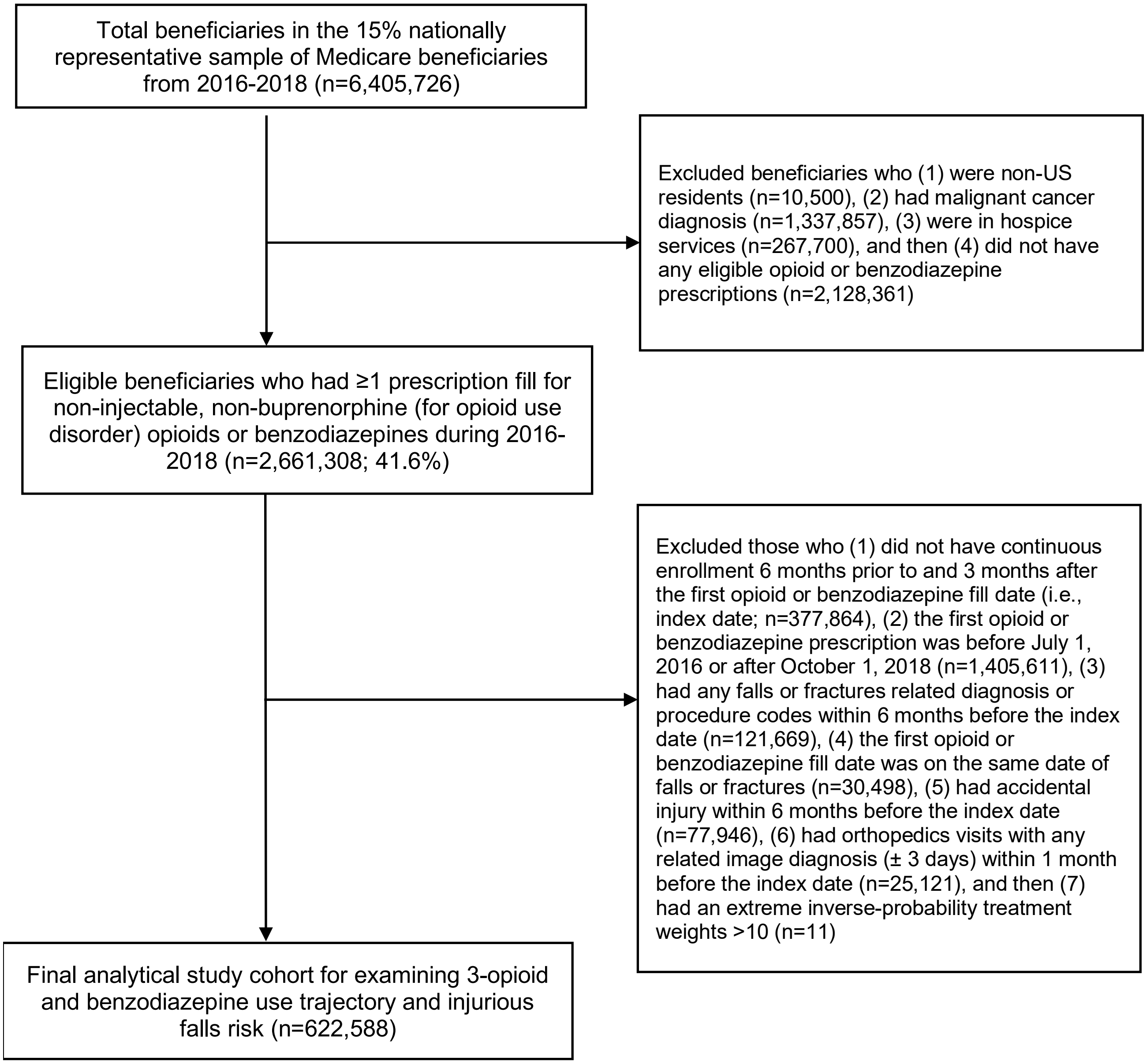

Figure S1. Sample Size Flowchart

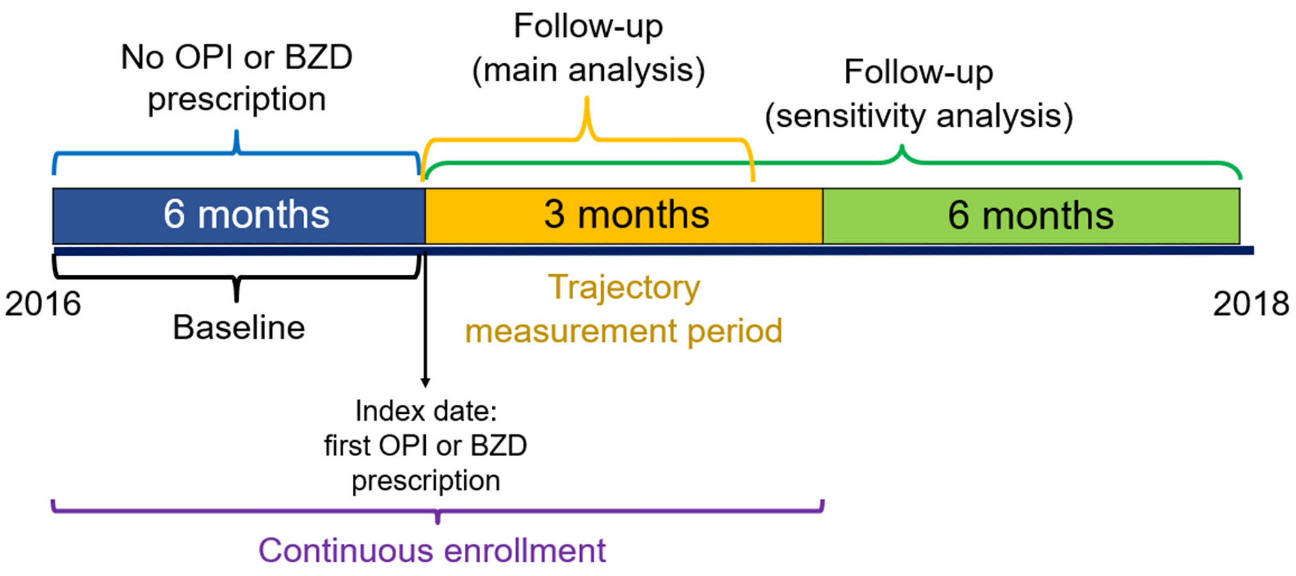

Figure S2. Study Design Diagram

Abbreviations: BZD, benzodiazepines; OPI, opioids

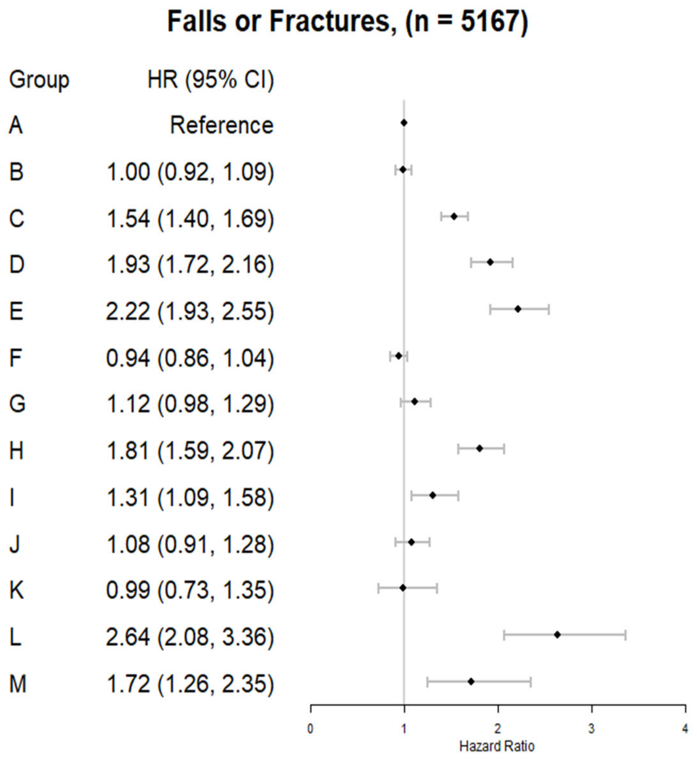

A: Very-low OPI-only (rapid decline) (n=279,263, 44.9% of the cohort)
B: Low OPI-only (rapid decline) (n=93,703, 15.1%)
C: Very-low OPI-only (delayed decline) (n=47,851, 7.7%)
D: Low OPI-only (n=24,952, 4.0%)
E: Moderate OPI-only (n=14,225, 2.3%)
F: Very-low BZD-only (n=71,715, 11.5% of the cohort)
G: Low BZD-only (declining) (n=28,109, 4.5%)
H: Low BZD-only (stable) (n=19,230, 3.1%)
I: Moderate BZD-only (n=13,013, 2.1%)
J: Very-low OPI; Very-low BZD (n=17,750, 2.9% of the cohort)
K: Very-low OPI / Very-low BZD (n=5,601, 0.9%)
L: Very-low OPI; low BZD (stable) (n=3,729, 0.6%)
M: Low OPI; Low BZD (n=3,447, 0.6%)

Figure S3. Trajectories of Opioid and Benzodiazepine Utilization Patterns and Risk of Injurious Falls: Sensitivity Analyses including Beneficiaries with Injurious Falls During the 6-month Trajectory Period

Abbreviations: BZD, benzodiazepine; CI, confidence intervals; DME, diazepam milligram equivalent; HR, hazard ratio; MME, morphine milligram equivalent; OPI, opioid; OPI-BZD, concurrent opioid and benzodiazepine use; SDD, standardized daily dose

To facilitate the labeling of opioid and benzodiazepine dose levels for each trajectory, we defined opioid dosage use as: very-low- (SDD <25 MME), low- (25–50 MME), moderate- (51–90 MME), high- (91–150 MME), and very-high-dose (>150 MME). Similarly, we defined BZD dosage use as very-low- (<10 DME), low- (10–20 DME), moderate- (21–40 DME), high- (41–60 DME), and very-high-dose (>60 DME).
